# Work Productivity Impairment in Persons with Inflammatory Bowel Diseases: A Systematic Review and Meta-analysis

**DOI:** 10.1093/ecco-jcc/jjae057

**Published:** 2024-04-22

**Authors:** Michael Youssef, Nariman Hossein-Javaheri, Tedi Hoxha, Christina Mallouk, Parul Tandon

**Affiliations:** Department of Internal Medicine, University of Toronto, Toronto, ON, Canada; Department of Internal Medicine, University at Buffalo, Buffalo, NY, USA; Department of Internal Medicine, University of Toronto, Toronto, ON, Canada; Faculty of Medicine, University of Ottawa, Ottawa, ON, Canada; Division of Gastroenterology and Hepatology, University Health Network, University of Toronto, Toronto, ON, Canada

**Keywords:** Inflammatory bowel disease, work productivity, WPAI

## Abstract

**Background and Aims:**

The impact of inflammatory bowel disease [IBD] on work productivity remains unclear. In this systematic review and meta-analysis, we quantify work-related outcomes and employment data among persons with IBD.

**Methods:**

A systematic literature search was conducted in MEDLINE, EMBASE, the Cochrane library, Scopus, ProQuest, and clinicaltrials.gov from inception to February 2023, to identify studies on work productivity in persons with IBD aged > 18 years. Work productivity was defined primarily by the Work Productivity and Activity Impairment [WPAI] questionnaire which includes absenteeism, presenteeism, overall work impairment, and non-work activity impairment. In addition, we included data on employment, sick leaves, disability pensions, and indirect costs due to productivity loss. Pooled effect analysis was conducted using a random-effects model for pooled estimates of continuous and proportional data with 95% confidence intervals.

**Results:**

Among all patients with IBD, the pooled estimates were 16.4% for absenteeism, 35.9% for presenteeism, 39.4% for overall work impairment, and 46.0% for non-work activity impairment. Indirect costs from overall work impairment were 5131.09 euros/patient/year. Only two-thirds of IBD patients were employed, and one in three lost their jobs due to IBD. Among those employed, 39.5% report sick days, 21.3% report work disability, and 12.3% receive disability pensions. Most studies demonstrate clinically meaningful improvements in work productivity with medical and/or surgical therapies.

**Conclusion:**

Persons with IBD experience significant work impairment and associated indirect costs. This highlights the need for appropriate workplace accommodations and timely medical therapy to alleviate the burden of disease and improve work outcomes.

## Graphical Abstract

**Graphical Abstract:**
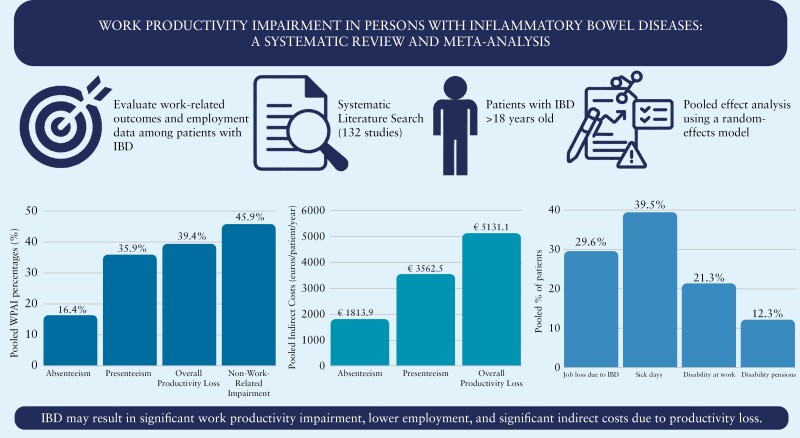

## 1. Introduction

Inflammatory bowel diseases [IBD], which include Crohn’s disease [CD] and ulcerative colitis [UC], are chronic disorders that affect approximately 6.8 million people globally.^1^ IBD is associated with debilitating symptoms including diarrhoea, abdominal pain, and significant fatigue. Individuals experiencing severe disease activity and flares may also require hospitalisation and surgical intervention.^2^ Therefore, the natural history of the disease, often recurring and fluctuating, coupled with these burdensome symptoms, can result in significant impairment in individuals’ quality of life [QOL] and functioning in society.

The average age of disease onset is between 31–34 years, coinciding with peak years of professional life.^3^ Compared with the general population, individuals with IBD have higher rates of unemployment, sick leave, and work disability.^4^ Though the effects of disability in IBD have been increasingly documented, it is only recently that standardised measures of impairment have been applied in this population. One such measure is the Work Productivity and Activity Impairment score [WPAI], which measures work time missed and work and activity impairment due to a specified health problem during the past 7 days.^5^ The WPAI has been validated in both CD and UC, among other disease states.^6^

Despite the development of this tool, there remains a significant variability in data and outcome definitions regarding work impairment in persons with IBD. This has limited prior attempts to conduct meta-analyses to accurately quantify the extent of work impairment. In addition, the indirect costs associated with productivity losses have yet to be quantified, or reported, systematically. As such, it becomes challenging to clearly describe the socioeconomic burden of the disease and its impact on patients in their workplaces. This research gap is crucial to better understand the psychosocial aspects of the disease and to advocate for workplace accommodations that may mitigate the work disability experienced by patients with IBD. In this systematic review and meta-analysis, we aim to closely quantify the impact of IBD on work productivity and the indirect costs, using the standardised WPAI questionnaire, and to review medical and surgical interventions that may affect work-related outcomes in IBD.

## 2. Materials and Methods

This systematic review and meta-analysis aimed to determine the impact of IBD on work productivity. The study was conducted according to the Preferred Reporting Items for Systematic Reviews and Meta-Analyses [PRISMA] statement guidelines, and a priori registered on PROSPERO [ID: CRD42023399459].

### 2.1. Search strategy and eligibility criteria

With the assistance of a trained medical librarian, we conducted a literature search on MEDLINE, EMBASE, and Cochrane electronic databases. In addition, we searched Scopus, ProQuest, and clinicaltrials.gov for abstracts, conference presentations, and other grey literature. These databases were searched from inception to February 2023 [full search strategy included as Supplementary Material]. To complement our database search, we also screened the reference lists of the included studies and review articles to identify any additional eligible studies.

We included full texts and abstracts of observational studies and clinical trials that reported work productivity outcomes and indirect costs in adult IBD patients [18 years and older]. Exclusion criteria included non-IBD studies, paediatric populations [<18 years], studies with no work-related outcomes, narrative and systematic review articles, articles with no full text available, non-English studies, and uncompleted studies. Studies with duplicate patient populations were excluded after including the most recent study on that population. Studies were also excluded if employment data was not a study outcome. In addition, we excluded studies where work impairment was not directly related to IBD, but rather solely a consequence of inconveniences with medical treatment [eg, biologic infusions], surgical interventions [eg, post-colectomy], caregiver burnout, or layoffs during the COVID-19 pandemic. Otherwise, we included these studies if they reported IBD-specific work productivity outcomes. Last, we excluded studies that reported data as comparisons between groups or as ‘mean changes’ before and after an intervention with no absolute values. Where there were missing data, we attempted to contact the corresponding authors of the primary studies.

### 2.2. Study selection

Four reviewers [MY, NJ, TH, and CM] independently performed the initial title and abstract screen and selected abstracts that met inclusion criteria for full text review. The reviewers then independently reviewed the full length manuscripts [or abstracts] and included eligible studies in the final review. Any discrepancies were resolved through consensus agreement.

### 2.3. Outcome definitions

The primary outcome of this study was to determine the impact of IBD on work productivity outcomes defined primarily by WPAI. The questionnaire generates percentages [0–100%] relating to the past 7 days of work, quantifying absenteeism [percentage of time missed from work], presenteeism [percentage of impaired functionality at work despite physical presence], overall work impairment [percentage of absenteeism + presenteeism], and non-work activity impairment, with higher scores indicating greater impairment.^5^ Secondary outcomes included other non-WPAI work-related outcomes as aggregated by each study, including sick leaves, number of working days missed, work disability due to IBD, disability pensions, lost jobs due to IBD, and WPAI-related indirect costs. These indirect costs were abstracted directly from studies and reported exactly as calculated in each individual study.

### 2.4. Data collection

The four reviewers independently completed data extraction using a standardised data collection sheet that was designed a priori. Data collected included: [a] study characteristics such as primary author, year of publication, nature of study [full text vs abstract], study design [prospective vs cross-sectional, and observational vs interventional]; [b] sample size of patients included with IBD [UC and CD]; [c] patient characteristics including age, sex, disease severity and activity; [d] employment data; [e] work-related outcomes defined primarily by WPAI and other non-WPAI outcomes as described above.

### 2.5. Data analysis

Meta-analysis using the continuous random-effects method was conducted for continuous outcomes to calculate the pooled mean effect sizes with 95% confidence intervals [CI] for WPAI outcomes and indirect costs. For studies that only reported data as means with CI, standard deviation [SD] was estimated using the formula SD= √ N x [upper–lower limit of CI]/3.92, where N is the sample size for which the data are reported.^7,8^ Binary random-effects method was used to calculate pooled incidence rates with 95% CIs for proportional outcomes. For interventional studies with multiple data points, we used the baseline employment and WPAI data [ie, pre-intervention data] for our meta-analysis. We then conducted subgroup analyses by IBD subtype [CD vs UC], and used mean differences and odds ratios [ORs] to compare patients with moderate/severe IBD vs those in remission or with mild IBD as defined by each individual study. Forest plots were generated for these comparisons where applicable. A pooled analysis was not conducted for interventional studies, given the significant variability in the interventions studied and the differences in data reporting between studies [absolute values vs mean changes].

To explore sources of heterogeneity, sensitivity analyses according to study type [cohort vs cross-sectional] and manuscript type [full length vs abstract] were performed. All summary estimates were determined by DerSimonian-Laird random-effects models. Between-study heterogeneity was assessed by the I^2^ statistic. An I^2^ > 50% suggested substantial heterogeneity.^9^ All statistical analyses were performed using OpenMeta version 10.12.^10^

### 2.6. Study quality assessment

Risk of bias for full text cross-sectional studies was assessed using the Appraisal tool for Cross-Sectional Studies [AXIS] tool.^13^ Full-text cohort and case-control studies were assessed using the National Institutes of Health [NIH] tool.^11^ Last, the Cochrane risk-of-bias tool [ROB] was used to assess full-text randomised control trials [RCTs].^12^

## 3. Results

**Figure 1:**
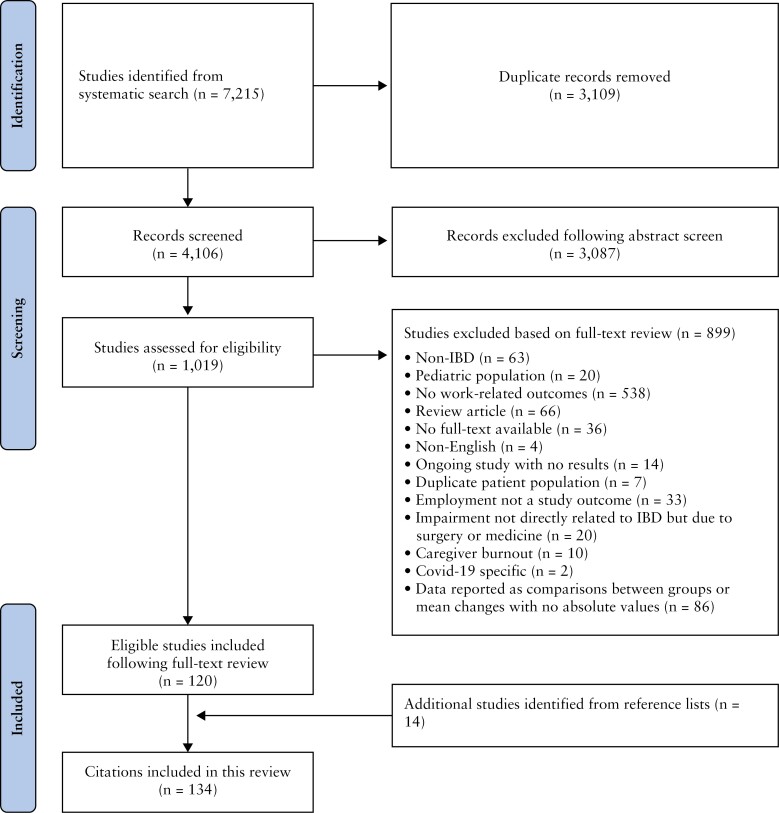
PRISMA flowchart.

Four thousand one hundred and six references were eligible for title and abstract screening [Figure 1]; 1019 studies were reviewed and 899 were excluded, leaving 120 studies eligible for inclusion. After reviewing the reference lists of included studies, 14 additional studies were included, resulting in 134 studies included in this review. This study included 96 full text articles and 38 abstracts. There were 105 observational studies [Table 1]^13–117^ and 29 interventional studies.^118–146^ The study characteristics and outcomes of these observational and interventional studies are included in Supplementary Tables S1 and S2, respectively.

**Table 1:** Observational studies—baseline characteristics.

Study	Country of study	Sample size	%Male, female	Mean age ± SD	Employment [%]	WPAI reported?	Indirect costs reported?	Miscellaneous outcomes reported?
Ding 2022^13^	USA	Total = 563 [CD = 281, UC = 282]	CD—M:52.3, F:47.7 UC—M:55.7, F:44.3	CD: 40.0 ± 12.1 UC: 40.5 ± 12.1	-	✓	✓	-
Decker 2022^14^	Czech Republic	Total = 161 [CD = 102, UC = 59]	M:41.6, F:58.4	41.2	-	✓	✓	✓
Holko 2022^15^	Belgium, Bulgaria, Cyprus, Czech Republic, Denmark, Greece, Hungary, Italy, Poland, Portugal, Romania, Spain	Total = 3687 [CD = 1930, UC = 1693, IC = 63]	M:34, F:66	43.03 ± 13.76	67	✓	✓	✓
Varma 2022^16^	USA	CD = 403	M:26.5, F:73.5	49.5 ± 15.9	64.3	-	-	✓
Paulides 2022^17^	The Netherlands	Total = 229 [CD = 155, UC = 66, IC = 8]	M:31, F:69	38.0 ± 17	66.8	✓	-	✓
Viazis 2022^18^	Greece	UC = 95	M:47, F:53	42 ± 13.69	-	-	-	-
Paulides 2020^19^	New Zealand	Total = 123 [CD = 83, UC = 40]	M:41, F:59	42.5 ± 13.5	100	-	-	✓
Topal 2020^20^	Turkey	Total = 180 [CD = 115, UC = 86]	M:57.5, F:42.5	-	-	-	-	✓
Yamabe 2019^21^	Japan	Total = 441	M:61.7, F:38.3	48.2 ± 14.8	66.2	✓	-	✓
Kawalec 2018^22^	Poland	UC = 147	M:46.94, F:53.06	39.0 ± 13.4	64.6	✓	✓	✓
Sciberras 2022^23^	8 European centres [not specified] + Israel	Total = 585 [CD = 363, UC = 222]	M:53, F:47	-	Total [*n* = 585]: 74.5 UC [*n* = 222] = 79.9 CD [*n *= 363] = 71.3	-	-	✓
vanGennep 2021^24^	The Netherlands	Total = 510 [CD = 268, UC = 242]	M:41, F:59	-	-	✓	✓	✓
Ruiz-Casas 2021^25^	Denmark, Norway, Poland, Romania, and Turkey	Total: 299 [Remission/mild = 1131, Moderate/severe = 1835]	Total—M:55, F:45 Remission/mild—M:53, F:47 Moderate/severe—M:56, F:44	Total: 47 ± 15 Remission/mild: 48 ± 15 Moderate/severe: 46 ± 15	-	✓	✓	-
Rankala 2021^26^	Finland	Total = 320 [CD = 102, UC = 218]	M:50.6, F:49.4	46.2	100	-	✓	-
Khalili 2020^27^	Sweden	Prevalent: 29,879 [CD = 10117, UC = 19762] Incident: 12,687 [CD = 4028, UC = 8659]	Prevalent—M:51.4, F:48.6 Prevalent—M:48.9, F:51.1	Prevalent: 50.1 ± 17.6 Incident: 45.6 ± 19.1	Prevalent CD: 80 Prevalent UC: 86	-	✓	✓
Yu 2021^28^	China	Total = 3000 [CD = 1922, UC = 973]	M:59.6, F:40.4	34	54.9	-	-	✓
deSaBritoFroes 2021^29^	Brazil	Total = 413	M:45, F:55	39.4	-	-	-	✓
Walter 2020^30^	Austria	Total = 510 [CD = 345, UC = 165]	M:26.3, F:73.3	40.4 ± 13.1	63.9	✓	✓	✓
Manceur 2020^31^	USA	Total = 6715	M:45.7, F:54.3	44.8	-	-	✓	-
Moon 2020^32^	South Korea	UC = 355	M:59.2, F:40.8	-	48.6	✓	-	-
Chao 2019^33^	Canada	Total = 207 [CD = 144, UC = 63]	M:42.5, F:57.5	-	79.2	✓	-	✓
Parra 2019^34^	Brazil	Total = 407 [CD = 264, UC = 143]	CD—M:45.8, F:54.2 UC—M:43.4, F:56.6	CD: 42.9 ± 13.0 UC: 45.9 ± 13.8	CD: 44.3 UC: 53	✓	-	✓
Christiansen 2019^35^	Denmark	Total = 185 [CD = 78, UC = 107]	CD—M:55.1, F:44.9 UC—M:43.9, F:56.1	-	CD: 73.3 UC: 74	✓	-	✓
Pillai 2019^36^	Switzerland	Total = 2365 [CD = 1353, UC = 1012]	CD—M:46, F:54 UC—M:52, F:48	CD: 41 ± 15 UC: 43 ± 14	73	-	✓	✓
LeBerre 2019^37^	France	Total = 1410 [CD = 874, UC = 493]	M:24, F:76	38 ± 10.1	80	✓	-	✓
Gonczi 2019^38^	Canada	Total = 525 [CD = 374, UC = 151]	M:41.7, F: 52.9	-	73	✓	-	-
Everhov 2019^39^	Sweden	Total = 2015 [CD = 1920, UC = 6, IC = 89]	M:50, F:50	-	-	-	-	✓
Everhov 2018^40^	Sweden	CD = 20638	M:48, F:52	-	-	-	-	✓
Spekhorst 2017^41^	The Netherlands	Total = 2794 [CD = 1740, UC = 1054]	CD—M:37, F:63 UC—M:47, F:53	-	CD: 56 UC: 63	-	-	✓
Kamat 2017^42^	India	Total = 84 [CD = 25, UC = 59]	CD—M:80, F:20 UC—M:71, F:29	CD: 21 ± 5.1 UC: 37.3 ± 4.4	CD: 72 UC: 78	-	✓	✓
Williet 2017^43^	France	Total = 1185 [CD = 721, UC = 462]	Total—M:38.5, F:61.5 CD—M:37.9, F:62.1 UC—M:39.7, F:60.3	-	Total: 59.4 CD: 60.5 UC: 57.9	✓	-	✓
Holko 2016^44^	Poland	CD = 200	M:42.2, F:57.8	31.80 ± 10.41	Total: 60 Active: 55.2 Remission: 64.5	-	✓	✓
DeBoer 2016^45^	The Netherlands	Total = 202 [CD = 128, UC = 74]	M:43, F:57	41 ± 12	61	-		
Aldeguer 2016^46^	Spain	UC = 285	M:51.2, F:49.8	44.5 ± 15.6	67	-	✓	✓
Vester-Andersen 2015^47^	Denmark	Total = 379 [CD = 155, UC = 224]	CD—M:44.5, F:55.5 UC—M:52.2, F:47.8	-	CD: 92.1 UC: 88.7	-	-	✓
Zand 2015^48^	USA	Total = 440 [CD = 221, UC = 219]	M:49.8, F:50.2	-	64.4	✓	✓	✓
Cohen 2015^49^	USA	UC = 4314	M:63.6, F:36.4	45.1	-	-	✓	✓
vanderHave 2015^50^	The Netherlands	Total = 1108 [CD = 554, UC = 424, IC = 130]	CD—M:42, F:58 UC—M:55, F:45	CD: 55 UC: 56.4	-	-	-	✓
Michael 2014^51^	Hungary	Total = 443 [CD = 260, UC = 183]	CD—M:47, F:53 UC—M:43.7, F:56.2	CD: 35 ± 11.3 UC: 40.7 ± 14.1	48	✓	✓	✓
Lonnfors 2014^52^	25 countries [not specified]	Total = 4670 [CD = 2895, UC = 1774]	M:33, F:66	35	-	-	-	✓
vanderValk 2014^53^	The Netherlands	Total = 2282 [CD = 1373, UC = 909]	CD—M:34.3, F:65.7 UC—M:43.8, F:56.2	CD: 44.1 ± 11.8 UC: 46.1 ± 11.4	CD: 53 UC: 66.6	-	-	✓
Gibson 2014^54^	Australia	UC = 175	M:47.4, F:52.6	41.7 ± 15.1	-	✓	-	✓
Siebert 2013^55^	Switzerland	Total = 1187 [CD = 699, UC = 488]	CD—M:46.1, F:53.9 UC—M:49.8, F:50.2	CD: 41.8 ± 14.7 UC: 42.6 ± 13.9	-	-	-	✓
Gunnarsson 2013^56^	USA	Total = 200	M:46, F:54	42.95	-	-	✓	✓
Hoivik 2013^57^	Norway	Total = 516 [CD = 341, UC = 160]	CD—M:50.6, F:49.4 UC—M:49.6, F:50.4	UC: 45.6 CD: 38.1	-	-	-	✓
Ramos 2015^58^	Spain	Total = 293 [CD = 151, UC = 142]	CD—M:54, F:46 UC—M:53, F:46	CD: 43.1 ± 11 UC: 48 ± 10.2	73	-	-	✓
Vaizey 2014^59^	UK	UC = 173	M:44.5, F:55.5	47	Full-time: 42.8, Part-time:13.3 Active: 58.5%, Remission: 54%	✓	-	✓
Viazis 2013^60^	Greece	Total = 1181 [CD = 539, UC = 642]	CD—M:52, F:48 UC—M:52, F:48	-	-	-	-	✓
Benedini 2012^61^	Italy	CD = 162	M:50, F:50	43	-	-	✓	-
Zhou 2010^62^	China	Total = 92 [CD = 52, UC = 40]	CD—M:67.55, F:32.5 UC—M:55.8, F:44.2	CD: 35.7 ± 11.6 UC: 45 ± 16.7	CD: 75 UC: 90.4	-	-	✓
Gibson 2008^63^	USA	Total = 15539 [CD = 6569, UC = 8970]	CD—M:43.26, F:56.74 UC—M:46.38, F:53.62	CD: 43.61 UC: 45.28	-	-	✓	-
Stark 2006^64^	Germany	Total = 483 [CD = 241, UC = 242]	CD—M:35, F:65 UC—M:45, F:55	CD: 41 ± 11 UC: 43 ± 12	CD: 63 UC: 67	-	✓	✓
Bernklev 2006^65^	Norway	Total = 495 [CD = 334, UC = 161]	M:51, F:49	41.2 ± 13.7	CD: 76.4 UC: 81.4	-	-	✓
Boonen 2002^66^	The Netherlands	Total = 680 [CD = 282, UC = 359, IU = 39]	M:45.7, F:54.3	41 ± 11.4	CD: 71.6 [M], 56.2 [F] UC: 74.9 [M], 62.4 [F]	-	-	✓
Bernstein 2001^67^	Canada	Study A = 2476 [CD = 1231, UC = 1245] Study B = 80 [CD = 33, UC = 47]	Study A—M:43.4, F:56.6 Study B—M:48.2, F:51.8	Study A: 42 [MEDIAN]	Study A: All IBD [M, *N* = 1074]: 79.9% All IBD [F, *N *= 1402]: 51.5% CD [M, *N* = 495]: 78.5% CD [F, *N *= 736]: 49.2% UC [M, *N *= 579]: 80.5% UC [F, *N* = 666]: 53.4%	-	-	✓
Sorensen 1987^68^	Denmark	CD = 106	-	44	65	-	-	✓
Sikirica 2022^69^	USA, France, Germany, Italy, Spain, and UK	CD = 2354	M:51.4, F:48.6	39	-	✓	-	-
Tiankanon 2021^70^	Thailand	Total = 209 [CD = 103, UC = 106]	M:49.3, F:50.7	47.3 ± 15.7	-	✓	-	-
Humberto 2021^71^	USA [Puerto Rico]	Total = 120 [CD = 91, UC = 29]	M:49.2, F:50.8	27.0 ± 9.71	69.17		-	✓
Wong 2020^72^	USA	UC = 697	M:42.2, F:57.8	Mild: 51.36 ± 16.91 Moderate/severe: 43.76 ± 14.84	Mild: 56.3 Mod/sev: 62.7	✓	-	✓
Armuzzi 2019^73^	Italy	CD = 552	M:51, F:49	41	54	✓	-	✓
Limdi 2019^74^	Not specified	UC = 1649	-	-	-	✓	-	-
Carels 2019^75^	Belgium	CD = 18	M:67, F:33	25 ± 2.3	-	✓	-	-
Raimundo 2018^76^	USA	Total = 1020	M:45, F:55	46	-	✓	✓	-
Armuzzi 2018^77^	USA, France, Germany, Italy, Spain, UK	UC = 1037	M:55.6, F:44.4	39.2 ± 13.8	-	✓	-	-
Sebastian 2018^78^	UK	UC = 52	M:50, F:50	40 ± 13.5	74	✓	-	-
Aiello 2018^79^	Italy	UC = 77	M:53.3, F:46.7	47.1 ± 13	49.4	✓	-	✓
DeLima 2018^80^	Brazil	CD = 95	M:48.5, F:51.5	41	-	-	-	✓
Hellstrom 2017^81^	Sweden	Total = 1843 [CD = 698, UC = 1145]	-	-	-	-	✓	✓
Ghosh 2017^82^	Not specified	UC = 1816	M:54.1, F:45.9	38.5 ± 14.6	-	✓	-	✓
Ganz 2016^83^	USA	CD = 539	M:46.6, F:53.4	48.2	57	-	✓	✓
KatzAvitan 2016^84^	Israel	Total = 405 [CD = 255, UC = 150]	-	CD: 40.4 ± 14.9 UC: 52.8 ± 15.8	CD: 61.6 [active: 52, inactive: 73] UC: 59.3 [active: 52, inactive: 63]	✓		
Camacho 2016^85^	Spain	Total = 127	-	-	53.5	✓	-	✓
Schwartz 2016^86^	Israel	CD = 597	M:42.6, F:57.4	37.9 ± 11.2	M: 72.3, F: 54.9	✓		
VanAssche 2015^87^	11 European countries [not specified]	UC = 250	M:59, F:41	46.6 ± 16.3	-	✓		
Huascar 2015^88^	Not specified	Total = 292 [CD = 151, UC = 141]	M:43, F:47	45 ± 11	-	-		
Carpio 2015^89^	Spain	UC = 436	M:53, F:47	46 ± 13	-	-		
Geccherle 2015^90^	Italy	CD = 47	-	-	-	✓		
Piercy 2015^91^	France, Germany, Italy, Spain, and USA	Total = 2065 [CD = 1084, UC = 981]	CD—M:49.5, F:50.5 UC—M:49.2, F:50.8	CD: 39.6 UC: 39.4	-	✓	-	-
Miller 2014^92^	USA	Total = 68 [CD = 27, UC = 41]	-	-	72	-	-	✓
Wladysiuk 2014^93^	Poland	CD = 464	-	-	56	✓	-	-
Zand 2014^94^	USA	Total = 365	-	-	-	✓	-	✓
Kroeker 2012^95^	Canada	Table 1: Total = 202 [CD = 129, UC = 73] Table 2: Total = 138 [CD = 83, UC = 55]	CD—M:38.8, F:61.2 UC—M:39.7, F:60.3	CD: 24.6 ± 0.35 UC: 24.8 ± 0.33		-	-	✓
Cohen 2012^96^	USA	UC = 5157	M:63.7, F:36.3	48	100	-	✓	✓
Wilson 2012^97^	Not specified	Total = 4990 [CD = 3143, UC = 1647]	-	-	-	-	-	✓
Naim 2011^98^	USA	Total = 534	M:33, F:67	-	55	✓	-	✓
Gomollon 2011^99^	Spain	CD = 1688	M:49, F:51	42.5 ± 11.2	57	✓	-	✓
Kane 2009^100^	USA	CD = 247	M:38.5, F:61.5	43	Full-time: 46 Part-time: 13	-	-	✓
Procaccini 2007^101^	USA	Total = 173	-	-	-	-	-	✓
Yan 2020^102^	China	Total = 891 [CD = 522, UC = 363, IC = 6]	Total—M:59, F:41 CD—M:61.5, F:38.5 UC—M:59.9, F:40.1	Total: 40 CD: 37 UC: 44	Employee—CD: 53.8, UC: 60.1, Total: 56.2 Self-employed—CD: 10.5, UC: 14.3, Total: 12.2	-	-	✓
Ueno 2017^103^	Japan	Total = 172 [CD = 83, UC = 84, IU = 1]	M:66, F:32	-	-	-	-	✓
VanDerValk 2014^104^	The Netherlands	Total = 2252 [CD = 1315, UC = 937]	CD—M:37.3, F:62.7 UC—M:51.4, F:48.6	CD: 47.8 ± 13.6 UC: 49.8 ± 13.3	CD: 53.6 UC: 61.2	-	✓	✓
Hendrikson 1980^105^	Denmark	UC = 122	M:43, F:57	-	71	-	-	✓
Binder 1985^106^	Denmark	CD = 185	M:40, F:60	-	-	-	-	✓
Nurmi 2013^107^	Finland	Total = 556 [CD = 153, UC = 365, IC = 37]	Total—M:47.5, F:52.5 CD—M:40.5, F:59.5 UC—M:51, F:49 IC—M:43.2, F:56.8	-	-	-	-	✓
Ghosh & Mitchell 2007^108^	UK	Total = 5636 [CD = 3025, UC = 2333]	Total—M:42.8, F:56.9 CD—M:40, F:59.7 UC—M:46, F:53.7	-	-	-	-	-
Stjernman 2011^109^	Sweden	Total = 505 [CD = 497, UC = 284]	CD—M:40.8, F:59.2 UC—M:55.6, F:44.4	CD: 46 UC: 46	-	-	-	✓
Longobardi 2003^110^	USA	Total = 187 [105 with symptoms in the past 12 months, 82 without symptoms in the past 12 months]	With symptoms—M:33.5 ± 5.6; F:66.5 ± 5.6 No symptoms—M:36.7 ± 5.5; F:63.3 ± 5.5	-	-	-	✓	✓
Longobardi 2003^111^	Canada	Total = 187	M:31.6, F:68.4	-	-	-	✓	✓
Mayberry 1992^112^	UK	CD = 58	-	31 ± 5	79	-	-	✓
Gazzard 1978^113^	Not specified	CD = 85	M:33, F:67	35 ± 6	-	-	-	✓
Juan 2003^114^	Spain	CD = 635	M:47.9, F:52.1	33.1 ± 11.9	48	-	✓	✓
Ananthakrishnan 2008^115^	USA	CD = 737	M:37.8, F:62.2	45.8	-	-	-	✓
Blomqvist 1997^116^	Sweden	Total = 77 [CD = 39, UC = 38]	-	-	-	-	✓	✓
Mesterton 2009^117^	Sweden	CD = 420	M:45.6; F:54.4	50.2 ± 15.1	-	-	✓	-

IBD, Inflammatory bowel disease; CD, Crohn’s disease; UC, ulcerative colitis; IC, indeterminate colitis; M, male; F, female.

### 3.1. WPAI work outcomes

Table 2 provides a summary of the pooled estimates for WPAI outcomes and related indirect costs for all patients with IBD, CD only, and UC only. Among all patients with IBD, the pooled estimates were 16.4% [95% CI 13.9-18.9] for absenteeism, 35.9% [95% CI 31.1-40.7] for presenteeism, 39.4% [95% CI 33.9-44.9] for overall work impairment, and 46.0% [95% CI 39.5-52.5] for non-work activity impairment [Table 2]. The pooled estimates for indirect costs from absenteeism, presenteeism, and overall work impairment were 1813.9 [95% CI 907.8-2720.2], 3562.5 [95% CI 1,409.4-5,715.6], and 5131.1 [95% CI 800.9-9461.3] euros/patient/year, respectively [Table 2]. We were unable to estimate the costs of non-work activity impairment in IBD patients, nor the costs of presenteeism and overall work impairment by IBD subtype.

**Table 2:** Pooled mean estimates of WPAI outcomes and indirect costs.

WPAI outcome	No. of studies	Pooled mean estimate [%] [95% CI]	Heterogeneity [I^2^]
Absenteeism			
Total IBD	27	16.4 [13.9-18.9]	95.5
CD only	13	18.9 [14.9-22.9]	93.3
UC only	13	16.5 [13.0-20.1]	95.4
Presenteeism			
Total IBD	27	35.9 [31.1-40.7]	98.8
CD only	13	41.3 [37.2-45.5]	94.9
UC only	13	33.6 [27.8-39.5]	98.3
Overall work impairment			
Total IBD	27	39.4 [33.9-44.9]	98.9
CD only	13	45.5 [40.0-50.9]	96.5
UC only	12	38.0 [30.9-45.2]	98.6
Non-work activity impairment			
Total IBD	24	46.0 [39.5-52.5]	99.4
CD only	12	52.6 [49.0-56.3]	95.7
UC only	11	40.8 [32.2-49.5]	99.3

Indirect costs^a^	No. of studies	Pooled mean estimate [95% CI] [euro/patient/year]	Heterogeneity [I^2^]
Absenteeism			
Total IBD	8	1,813.9 [907.8-2720.2]	98
CD only	3	1,541.2 [955.1-2127.4]	79.1
UC only	3	1,170.5 [395.8-1945.1]	97.7
Presenteeism			
Total IBD	6	3,562.5 [1,409.4-5,715.6]	99.3
Overall work impairment			
Total IBD	5	5,131.1 [800.9-9,461.3]	99.9

WPAI, Work Productivity and Activity Impairment questionnaire; IBD, inflammatory bowel disease; UC, ulcerative colitis; CD, Crohn’s disease.

^a^There were not enough studies to determine pooled estimates of indirect costs separately for CD and UC with regards to presenteeism and overall work impairment.

Three studies reported WPAI absenteeism and presenteeism as time [hours] rather than percentages. The pooled estimate for absenteeism was 4.1 h per week [95% CI 3.1-5.2] [I^2^ = 54.3%] of missed work time due to IBD, whereas the pooled estimate for presenteeism was 3.9 h per week [95% CI 2.2-5.6] [I^2^ = 98.0%] of time experiencing impairment while at work. Five studies reported mean annual sick days, with a pooled estimate of 23.9 working days [95% CI 12.5-35.2] [I^2^ = 97.4%] lost due to IBD.

**Figure 2:**
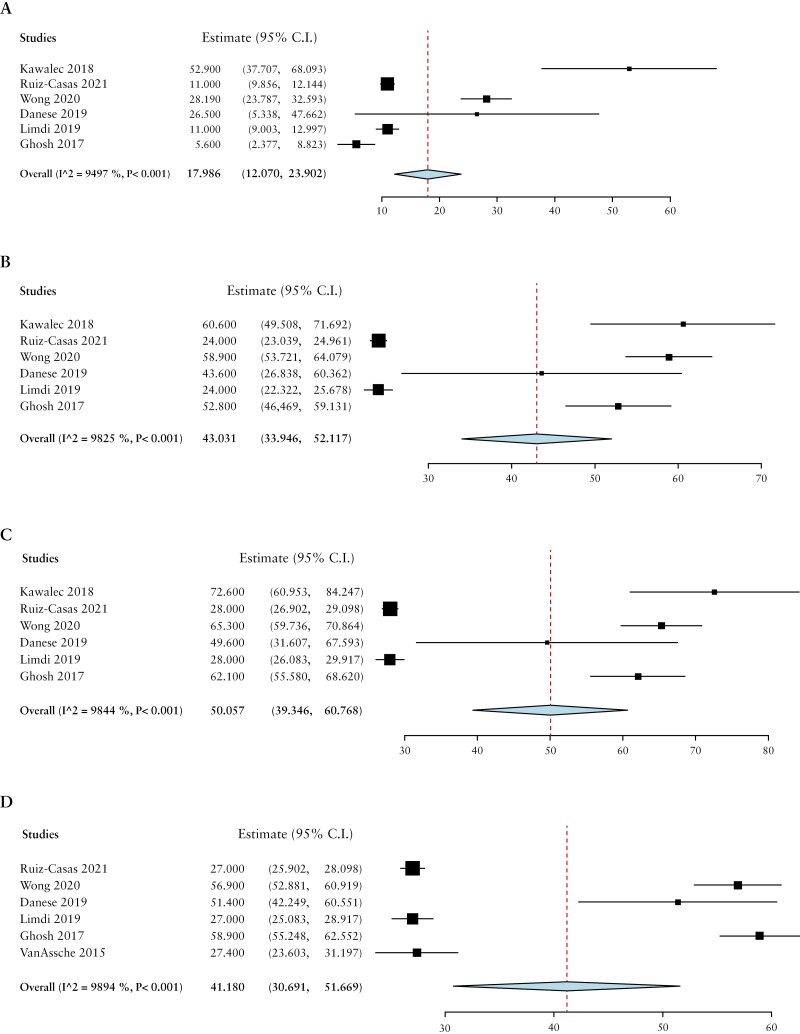
Comparison of A] absenteeism, B] presenteeism, C] total work impairment, D] non-work activity impairment, by mean differences based on disease activity.

Patients with active or moderate-severe disease had higher absenteeism [mean difference 18.0%, 95% CI 12.1-23.9], presenteeism [mean difference 43.0%, 95% CI 33.9-52.1], overall work impairment [mean difference 50.1%, 95% CI 39.3-60.8], and non-work activity impairment [mean difference 41.2%, 95% CI 30.7-51.7] than those in remission or with mild disease [Figure 2].

### 3.2. Non-WPAI work outcomes

The pooled estimate for employment among all patients with IBD was 65.6% [95% CI 61.9-69.3%] [Table 3]. Unemployment was considered as a separate outcome in most studies and not directly related to employment values. The pooled percentage of unemployment was 16.4% [95% CI 14.5-18.3] in all patients with IBD. Among all patients with IBD, 39.5% [95% CI 16.8-62.3] reported sick days, 21.3% [95% CI 16.6-26.0] reported disability at work specifically due to IBD, 12.3% [95% CI 10.7-13.9] reported receiving disability pensions, and 29.6% [95% CI 19.0-40.1] reported losing jobs due to IBD.

**Table 3:** Pooled proportions [percentages] of non-WPAI work-related outcomes.

Work outcome	No. of studies	Pooled percentage [95% CI]	Heterogeneity [I^2^]
Employment			
Total IBD	73	65.6% [61.9-69.3]	99.7
CD only	36	63.5% [57.9-69.0]	99
UC only	35	68.1% [64.9-73.3]	99.6
Unemployment^a^			
Total IBD	51	16.4% [14.5-18.3]	98.7
CD only	22	15.2% [11.8-18.6]	98
UC only	25	13.0% [10.2-15.8]	98.3
% reporting sick days			
Total IBD	25	39.5% [16.8-62.3]	99.9
CD only	10	28.5% [22.8-34.2]	99.3
UC only	15	34.8% [5.1-64.5]	99.9
% Disability due to IBD			
Total IBD	28	21.3% [16.6-26.0]	99.3
CD only	16	21.0% [14.6-27.4]	98.8
UC only	15	17.8% [12.0-23.6]	98.8
% Disability pensions			
Total IBD	23	12.3% [10.7-13.9]	96.7
CD only	10	14.4% [12.0-16.8]	95.2
UC only	10	9.7% [6.4%-13.0]	98.3
% losing jobs due to IBD	4	29.6% [19.0-40.1]	
Total IBD			98.8

WPAI, Work Productivity and Activity Impairment questionnaire; IBD, inflammatory bowel disease; UC, ulcerative colitis; CD, Crohn’s disease.

^a^Patient employment data was not solely limited to employment vs unemployment. Accordingly, unemployment was analyzed as a separate outcome [as opposed to 1-employment]

**Figure 3:**
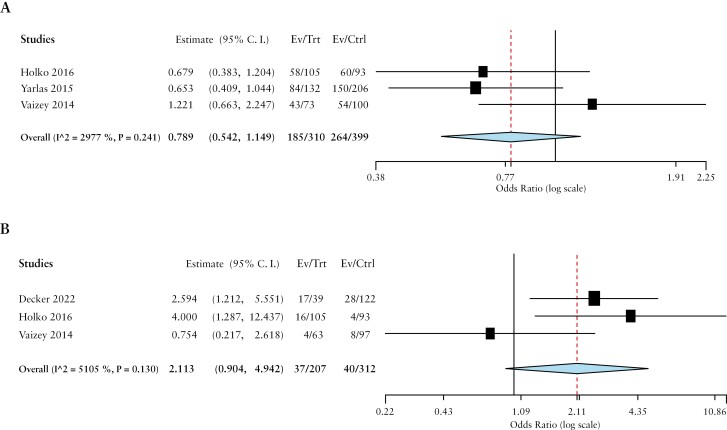
Comparison of A] employment and B] disability pensions by ORs based on disease activity.

With respect to disease activity, IBD patients with active disease had lower odds of being employed (OR 0.8, 95% CI 0.5-1.1 [I^2^ = 29.7%]) and higher disability pension rates (OR 2.1, 95% CI 0.9-4.9 [I^2^ = 51.0%]) than those in remission or with mild disease [Figure 3].

### 3.3. Interventional studies

Twenty nine interventional studies [26 medical and three surgical] were included in this systematic review. Most studies showed statistically significant and clinically meaningful differences in all WPAI subcategories with respect to medical interventions such as mesalamine, biologics, and/or small molecules. In studies on adalimumab, at different times of follow-up, the mean change in absenteeism ranged from -6.7 to -11.4, presenteeism from -14.5 to -24.5, total work impairment from -14.5 to -29.2, and non-work activity impairment from -16.7 to -27.2.^123,125^ Similar findings were observed with other biologics, such as infliximab and golimumab, as well as with small molecules.^118,129,133,135–137,139–143^ Three studies with surgical interventions were included and had conflicting results with regards to work outcomes.^144–146^ One study reported improved capacity to work with J-pouch surgery,^145^ whereas another study showed more sick days following colectomy.^144^ A third study investigated different surgical approaches and reported different morbidities and work impairment with each approach.^146^

### 3.4. Risk of bias

As per the AXIS tool, most cross-sectional studies were scored at 15/20 or above, suggesting overall moderate or high quality studies. Most cohort and case-control studies had a ‘good’ overall rating and some were rated as ‘fair’ as per the NIH tool. The risk of bias assessment as using the ROB tool revealed ‘some concerns’ in most of the included RCTs [Supplementary Tables S3–5].

## 4. Discussion

In this systematic review and meta-analysis, we demonstrate that two-thirds of patients with IBD report being employed and almost one in three lose their job specifically due to IBD. Among patients who are employed, almost half report taking sick days, a quarter experience disability at work, and one in 10 receives disability pensions. Furthermore, patients with IBD reported missing approximately 16% of their working hours [absenteeism] and experiencing significant functional impairment for almost half of their total working time [presenteeism]. Absenteeism and presenteeism related to IBD are significantly higher than in the general population, where health-associated absenteeism is reported to be within 1.5–3% and presenteeism around 23%.^147–149^ The overall productivity loss, through absenteeism and presenteeism, among patients with IBD is estimated around 40% of their working time. In addition, almost half of their non-work activity time is impaired due to IBD. Importantly, in using the WPAI questionnaire, we show a significantly high burden of disease on work productivity outcomes that are consistent and standardised across studies. This may clarify the true impact of the disease and highlight the need for evidence-based interventions to improve work productivity outcomes.

Persons with IBD experience significant challenges at work due to IBD-related symptoms including fatigue, chronic abdominal pain, and diarrhoea requiring frequent bathroom use.^150^ Up to 70% of patients may experience difficulty focusing at work and completing tasks, and many have shorter work days due to IBD symptoms.^92^ Additionally, the fluctuating nature of symptoms and unexpected flare-ups may lead to unplanned absences and difficulties committing to future work tasks or making career plans.^37,151^ Interestingly, IBD often affects absenteeism to a lesser extent than presenteeism,^43^ which is consistent with our results. This is important to note, given that productivity losses associated with presenteeism are significantly higher than those from absenteeism. One explanation is that patients may feel obliged to attend work for fear of financial repercussions or job insecurity, but continue to have debilitating symptoms at work, affecting their productivity.^152^ This may reflect patients’ fear of discussing their diagnosis at their workplace or requesting accommodations to feel comfortable at work.^150^

Previously published studies on work impairment in IBD have significant variability which has led to inconsistent findings. For instance, absenteeism has been estimated between 5% and 20%,^24,48^ and presenteeism and total work impairment have ranged from 20% to 65% and 20% to 50%, respectively.^24,34,43^ The variability in data might be due to residual confounding from underlying disease activity. Furthermore, studies have differed by proportion of patients on advanced IBD therapies, which in turn alters IBD activity and subsequently patients’ work productivity.^153^ We observed significant variability in the proportion of biologic use across the included studies, ranging from 0% to 94%. Furthermore, we noted significant variability in the definitions for work status and employment across studies. For example, some studies included any working individual with IBD [including students or stay-at-home parents], and others included only full-time, part-time, and/or self-employment. Finally, fluctuations within the labour market over time and between different countries may have also resulted in significant variability in overall employment and work outcomes in IBD.^154^

We also demonstrated that compared with those with UC, persons with CD have a greater degree of absenteeism, presenteeism, overall work, and total activity impairment. This is consistent with previous literature.^21,51,53^ Overall, CD patients face a greater degree of disability in multiple life domains compared with those with UC.^155^ These findings may be due to a greater systemic and psychological impact of CD.^65,156^ In addition, the incidences of chronic fatigue and depression are often greater among patients with CD, and these may subsequently interfere with work performance.^157,158^ Another important factor associated with poor work outcomes is disease activity. In particular, we observed higher WPAI scores among patients with active and moderate–severe disease, which is consistent with previous studies.^159,160^ Patients with more severe disease experience more significant work impairment, likely due to increased fatigue and poor health-related QOL.^161^In fact, twice as many patients with active IBD report fatigue, compared with those in remission.^162^ As such, disease activity is an important factor to consider when caring for patients with IBD.

We also reported significant indirect costs associated with decreased workplace productivity and sick leave in patients with IBD. In particular, we observed that the average annual indirect costs from total work impairment due to IBD was estimated to be greater than 5000 euros/patient/year. These costs are higher among those with a greater degree of absenteeism, among those living with CD compared with UC,^64,104,155,163,164^ and those with active inflammation.^24,165^ Overall, these findings suggest a high economic burden related to absenteeism and work productivity loss from IBD. Absenteeism may also be related to the lack of workplace accommodations, which makes the work environment challenging. Studies show that almost 90% of persons with IBD needed workplace accommodations, yet many find it difficult to ask or arrange for accommodations. This may certainly contribute to absenteeism and decreased productivity at work.^166^ In contrast, providing appropriate workplace accommodations such as flexible working hours and locations and employer benefits [eg, paid sick leave and health insurances] can certainly improve work productivity and mitigate many of these indirect costs.^167^

Medical and surgical interventions in IBD may reduce work impairment. We observed that most interventional studies demonstrated clinically meaningful and statistically significant improvements in work outcomes. This was consistent across studies using mesalamine, biologics, and/or small molecules.^119,131,141^ Furthermore, following effective treatment with anti-tumour necrosis factor [anti-TNF] therapy, patients reported significant improvement in all elements of the WPAI regardless of their initial level of disability.^123,129,135,136^ In contrast, the impact of surgical interventions on work impairment in IBD remains controversial.^41,145^ Whereas some patients’ productivity may improve after surgery,^145^ others may experience an increased risk of work-related disability and sick days due to post-surgical complications such as anastomotic leaks.^53,58^ Overall, medical and surgical therapies may improve work productivity among persons with IBD, although patient-specific factors should guide treatment decisions to determine the best therapeutic plan for each patient.

This study has several strengths. We employed a broad literature search which allowed us to identify a large number of eligible studies and approximate the burden of IBD across a number of jurisdictions worldwide. We used the WPAI questionnaire to standardise work productivity outcomes and accurately estimate effect sizes, despite the inherent limitations of the literature data. Additionally, we were able to delineate the association of disease subtype [CD vs UC], severity, and treatment modality [medical vs surgical] with work productivity outcomes. Despite this, the study has inherent limitations. First, there is significant heterogeneity across the IBD literature with regards to work productivity. This is likely due to significant variations in study populations, study design, definition of the underlying IBD diagnosis, and definitions of work outcomes across studies. For example, some studies defined IBD through self-reported questionnaires, and others used case history and endoscopic criteria. Similarly, employment was defined differently across studies as described above. Study results also varied across different geographical regions, reflecting differences in the workforce across countries. Furthermore, indirect costs were calculated based on the average wage and number of weeks worked per year, which may also be different across countries. Ultimately, this limits the generalisability of the study results when applying to specific jurisdictions. Second, there was a large number of excluded studies due to missing data or incomplete information. Furthermore, due to the lack of standardised outcome reporting in the literature, many studies did not include WPAI outcomes or compare them by disease severity. This limited our analyses to only a few eligible studies and may have led to selection bias. Additionally, we were unable to quantify the therapeutic effect of medical or surgical interventions on WPAI indices in a formal meta-analysis, due to the considerable heterogeneity in data reporting across studies. Overall, this highlights the need for standardisation of study designs and outcome definitions to guide future research in this important field.

## Conclusion

In conclusion, this study uses standardised tools to highlight the significant burden of IBD on work productivity. IBD type, disease severity, and medical therapy are all important factors that may affect work productivity outcomes. Future studies are needed to explore different workplace accommodations and their impact in improving work productivity for persons with IBD.

## Supplementary Data

Supplementary data are available at *ECCO-JCC* online.

jjae057_suppl_Supplementary_Material

## Data Availability

The data underlying this article are available and will be shared on reasonable request to the corresponding author.
