# Supplementary material for: Work Productivity Impairment in Persons with Inflammatory Bowel Diseases: A Systematic Review and Meta-analysis
Source: J Crohns Colitis. 2024 Apr 22;18(9):1486–504. doi: 10.1093/ecco-jcc/jjae057 (PMC11369077; doi:10.1093/ecco-jcc/jjae057)
Supplement: jjae057_suppl_Supplementary_Material [file jjae057_suppl_supplementary_material.docx]

**Supplementary Material (Search Strategy):**

**MEDLINE:**

| **#** | **Searches** | **Results** |
| --- | --- | --- |
| 1 | exp Inflammatory Bowel Diseases/ | 94588 |
| 2 | (inflammat* adj3 bowel* adj3 disease*).tw,kf. | 62044 |
| 3 | exp Crohn Disease/ | 43566 |
| 4 | Crohn*.tw,kf. | 55371 |
| 5 | exp Colitis, Ulcerative/ | 39741 |
| 6 | (ulcerative or colitis).tw,kf. | 88584 |
| 7 | or/1-6 | 161914 |
| 8 | (work* adj3 impairment*).tw,kf. | 3517 |
| 9 | (work* adj3 loss*).tw,kf. | 4627 |
| 10 | (productivity adj3 loss*).tw,kf. | 4482 |
| 11 | (job* adj3 loss*).tw,kf. | 1817 |
| 12 | (work* adj3 productivity).tw,kf. | 4980 |
| 13 | presenteeism.tw,kf. | 1763 |
| 14 | absenteeism.tw,kf. | 7183 |
| 15 | (work* adj5 quality of life).tw,kf. | 4274 |
| 16 | quality of work*.tw,kf. | 2885 |
| 17 | disabilit*.tw,kf. | 234850 |
| 18 | sick leave*.tw,kf. | 6391 |
| 19 | employment*.tw,kf. | 70509 |
| 20 | unemployment*.tw,kf. | 13716 |
| 21 | un-employment*.tw,kf. | 27 |
| 22 | (workplace* or (workplace* adj3 accommodat*)).tw,kf. | 52864 |
| 23 | (work place* or (work place* adj3 accommodat*)).tw,kf. | 2971 |
| 24 | (job* adj3 satisfaction*).tw,kf. | 11565 |
| 25 | pension*.tw,kf. | 7297 |
| 26 | indirect cost*.tw,kf. | 7057 |
| 27 | retirement*.tw,kf. | 17780 |
| 28 | (work* adj3 performance*).tw,kf. | 13843 |
| 29 | (productivity* adj3 impairment*).tw,kf. | 987 |
| 30 | or/8-29 | 426167 |
| 31 | 7 and 30 | 1037 |

**EMBASE:**

| **#** | **Searches** | **Results** |
| --- | --- | --- |
| 1 | exp inflammatory bowel disease/ | 193698 |
| 2 | (inflammat* adj3 bowel* adj3 disease*).tw,kf. | 104763 |
| 3 | exp Crohn disease/ | 109737 |
| 4 | Crohn*.tw,kf. | 98490 |
| 5 | exp ulcerative colitis/ | 93853 |
| 6 | (ulcerative or colitis).tw,kf. | 147051 |
| 7 | or/1-6 | 276510 |
| 8 | (work* adj3 impairment*).tw,kf. | 5979 |
| 9 | (work* adj3 loss*).tw,kf. | 7403 |
| 10 | (productivity adj3 loss*).tw,kf. | 7248 |
| 11 | (job* adj3 loss*).tw,kf. | 2190 |
| 12 | (work* adj3 productivity).tw,kf. | 8929 |
| 13 | presenteeism.tw,kf. | 3120 |
| 14 | absenteeism.tw,kf. | 11225 |
| 15 | (work* adj5 quality of life).tw,kf. | 6742 |
| 16 | quality of work*.tw,kf. | 3940 |
| 17 | disabilit*.tw,kf. | 340423 |
| 18 | sick leave*.tw,kf. | 8491 |
| 19 | employment*.tw,kf. | 98925 |
| 20 | unemployment*.tw,kf. | 16588 |
| 21 | un-employment*.tw,kf. | 34 |
| 22 | (workplace* or (workplace* adj3 accommodat*)).tw,kf. | 65389 |
| 23 | (work place* or (work place* adj3 accommodat*)).tw,kf. | 4790 |
| 24 | (job* adj3 satisfaction*).tw,kf. | 13791 |
| 25 | pension*.tw,kf. | 9734 |
| 26 | indirect cost*.tw,kf. | 11538 |
| 27 | retirement*.tw,kf. | 21318 |
| 28 | (work* adj3 performance*).tw,kf. | 17166 |
| 29 | (productivity* adj3 impairment*).tw,kf. | 2711 |
| 30 | or/8-29 | 592173 |
| 31 | 7 and 30 | 2635 |

**Cochrane Library:**

#1 MeSH descriptor: [Inflammatory Bowel Diseases] explode all trees

#2 (inflammat* bowel* disease*):ti,ab,kw

#3 MeSH descriptor: [Colitis, Ulcerative] explode all trees

#4 (ulcerative colitis):ti,ab,kw

#5 MeSH descriptor: [Crohn Disease] explode all trees

#6 (crohn* disease):ti,ab,kw

#7 #1 OR #2 OR #3 OR #4 OR #5 OR #6

#8 (work* NEAR impairment*):ti,ab,kw

#9 (work* NEAR loss*):ti,ab,kw

#10 (productivity* NEAR loss*):ti,ab,kw

#11 (job* NEAR loss*):ti,ab,kw

#12 (work* NEAR productivity*):ti,ab,kw

#13 (presenteeism):ti,ab,kw

#14 (absenteeism):ti,ab,kw

#15 (work* NEAR "quality of life"):ti,ab,kw

#16 (quality of work*):ti,ab,kw

#17 (disabilit*):ti,ab,kw

#18 (sick leave*):ti,ab,kw

#19 (employment*):ti,ab,kw

#20 (unemployment*):ti,ab,kw

#21 (workplace* NEAR accommodat*):ti,ab,kw

#22 (work place* NEAR accommodat*):ti,ab,kw

#23 (job* NEAR satisfaction*):ti,ab,kw

#24 (pension*):ti,ab,kw

#25 (indirect cost*):ti,ab,kw

#26 (retirement*):ti,ab,kw

#27 (work* NEAR performance*):ti,ab,kw

#28 (productivity* NEAR impairment*):ti,ab,kw

#29 #8 OR #9 OR #10 OR #11 OR #12 OR #13 OR #14 OR #15 OR #16 OR #17 OR #18 OR #19 OR #20 OR #21 OR #22 OR #23 OR #24 OR #25 OR #26 OR #27 OR #28

#30 #7 AND #29

**SCOPUS:**

(TITLE-ABS-KEY("work* impairment*" OR "work* loss*" OR "productivity loss*" OR "job* loss*" OR disabilit* OR presenteeism OR absenteeism OR employment* OR unemployment*)) AND (TITLE-ABS-KEY("inflammatory bowel disease*" OR "ulcerative colitis" OR "colitis" OR "Crohn* disease*")) AND ( LIMIT-TO ( LANGUAGE,"English" ) )

**ProQuest:**

noft("inflammatory bowel disease*" OR "ulcerative colitis" OR "colitis" OR "Crohn* disease*") AND noft("work* impairment*" OR "work* loss*" OR "productivity loss*" OR "job* loss*" OR disabilit* OR presenteeism OR absenteeism OR employment* OR unemployment*)

**Clinicaltrials.gov**

Inflammatory Bowel disease AND work

**Supplementary Table S1. Observational Studies - WPAI, Indirect Costs, and Miscellaneous Outcomes**

| Study | WPAI (% ± SD)  Absenteeism / Presenteeism / TWPI / OAI | Indirect Costs  (€/patient/yr unless otherwise specified) | Miscellaneous Outcomes |
| --- | --- | --- | --- |
| Ding 2022^13^ | CD - Remission (n=183): 0.95 / 11.69 / 12.38 / 12.36, Mild (n=59): 4.48 / 29.15 / 31.29 / 32.88, Mod/sev (n=39): 14.57 / 44.87 / 50.98 / 47.69  UC - Remission (n=83): 0.59 / 7.11 / 7.51 / 6.39, Mild (n=143): 2.17 / 18.58 / 19.71 / 18.81, Mod/sev (n=65): 11.85 / 37.08 / 41.93 / 41.08 | CD: USD $7169 (remission)-$29524 (mod/sev)/patient/yr  UC: USD $4348 (remission)-$24283 (mod/sev)/patient/yr | - |
| Decker 2022^14^ | 6.2 ± 18 / 13.2 ± 21.5 / 15.4 ± 23 / 19.3 ± 25.2 | Absenteeism: 1,168 ± 3607  Presenteeism: 2,111 ± 3421  Overall work impairment: 3,279 ± 5132  Total work impairment + disability pensions: 7,673 ± 9032 | 28% disability pensions |
| Holko 2022^15^ | **3.38 ± 21.71 (n=2455) / 5.27 ± 9.45 (n=2455) / - / - | Absenteeism: 3782 ± 17644.50  Presenteeism: 6457 ± 12146.15 | Unemployment: 9.2% |
| Varma 2022^16^ | - | - | 6.30% disability due to IBD  34.3% missed work due to CD (no formal score used) |
| Paulides 2022^17^ | 8.10 / 18.10 / 20.40 / - | - | Unemployment: 3% |
| Viazis 2022^18^ | - | - | - |
| Paulides 2020^19^ | - | - | 14% disability pensions |
| Topal 2020^20^ | - | - | 63.5% disability due to IBD  Unemployment: 40% |
| Yamabe 2019^21^ | 9.3 ± 22.1 (n=271) / 29.6 ± 28.7 (n=273) / 33.4 ± 32 (n=271) / 30 ± 28.8 (n=441) | - | Unemployment: 39.4% |
| Kawalec 2018^22^ | Total Working (n=95): 25.7 ± 39.5 / 37 ± 36.8/ 45.1 ± 40.8 / -  Remission (n=61): 11.1 ± 26.9 / 24.3 ± 32.5 / 30.2 ± 36.1 / -  Active (n=34): 52.9 ± 45.8 / 60.6 ± 33.0 / 72.6 ± 34.7 / - | All working patients (n=95) - Cost of absenteeism: 4161.1 ± 6573.9, Presenteeism: 3907.9 ± 5430.9, Total average indirect cost: 8069.0 ± 7638.8  Remission (n=61) - Cost of absenteeism: 1615.2 ± 3768.6, Presenteeism: 3684.4 ± 5244.6, Total average indirect cost: 5299.7 ± 6296.4  Active (n=34) - Cost of absenteeism: 8913.3 ± 8002.7, Presenteeism: 4325.1 ± 6077.0, Total average indirect cost: 13,238.4 ± 7196.9 | Number of workdays missed -  TOTAL WORKING (n= 95): 66.3 ± 101.9 days  Remission (n= 61): 27.4 ± 66.1 days  Active (n= 34): 138.8 ± 117.4 days |
| Sciberras 2022^23^ | - | - | Unemployment: 25.5% |
| vanGennep 2021^24^ | 5 ± 16 / 17 ± 23 / 21 ± 27 / - | Absenteeism: 1,738 ± 5505  Presenteeism: 5,478 ± 8629  Overall work impairment: 6,569 ± 9987 | 8% disability pensions  Severe absenteeism reported in 4%  Severe presenteeism reported in 16%  Severe overall WP loss reported in 19% |
| Ruiz-Casas 2021^25^ | Total: 9 ± 23 / 22 ± 20 / 25 ± 23 / 24 ± 23  Remission/Mild: 6 ± 19 / 18 ± 19 / 20 ± 21 / 18 ± 20  Mod/Sev: 11 ± 25 / 24 ± 21 / 28 ± 24 / 27 ± 24 | Sick leave -  Total: 630 ± 2689  Remission/Mild: 249 ± 1112  Mod/Sev: 875 ± 3311 | - |
| Rankala 2021^26^ | - | Absenteeism: 740.9 ± 3617.4  Presenteeism: 643.9 ± 2286.7 | - |
| Khalili 2020^27^ | - | Prevalent CD: $12,717 ± 24602 USD  Prevalent UC: $8,209 ± 20196 USD  Incident CD: $12,102 ± 23277 USD  Incident UC: $8,852 ± 20137 USD | Mean sick days - Prevalent CD: 19, Prevalent UC: 16, Incident CD: 29, Incident UC: 20  %patients reporting sick days - Prevalent CD: 20%, Prevalent UC: 18%  %disability pensions - Prevalent CD: 15%, Prevalent UC: 9%  #days on disability pension - Prevalent CD: 44 days, Prevalent UC: 25 days, Incident CD: 31 days, Incident UC: 24 days  Unemployment - Prevalent CD: 5%, Prevalent UC: 5% |
| Yu 2021^28^ | - | - | Labour force non-participation 6.5%  Unemployment: 6.9% |
| deSaBritoFroes 2021^29^ | - | - | 16.7% disability due to IBD |
| Walter 2020^30^ | **4.47 ± 11.9 / 2.82 ± 4.4 / 7.28 ± 12.9 / 4 ± 4.15 | Average work productivity loss: 7,411 ± 219  Relapse: 12,377  Remission: 6,040 | 35% patients reporting sick days  71.5% reported productivity loss while working |
| Manceur 2020^31^ | - | Total indirect costs: $5,490 USD  Absenteeism: $2,819 USD | - |
| Moon 2020^32^ | Total: 29.1 ± 36.9 / 37.8 ± 31.4 / 45.5 ± 32.7 / 46.4 ± 31.2  Mod: 27.3 ± 35.8 / 36.8 ± 30.3 / 44.1 ± 31.9 / 45.2 ± 31.0  Sev: 54.1 ± 43.5 / 62.2 ± 39.6 / 70.7 ± 37.8 / 61.2 ± 31.0 | - | - |
| Chao 2019^33^ | - / - / 21 (MEDIAN) / - | - | 47.2% of patients reported mild productivity loss (defined as 0-19%)  32.4% of patients reported mod productivity loss (defined as 20-49%)  20.4% of patients reported severe productivity loss (defined as >50%)  Unemployment: 14% |
| Parra 2019^34^ | CD (MEDIAN) -  Total: 0 / 10 / 20 / 30  Inactive/Mild: 0 / 10 / 19.7 / 20  Mod/sev: 4.7 / 10 / 30 / 50  UC (MEDIAN) -  Total: 0 / 0 / 5 / 30  Inactive/Mild: 0 / 0 / 0 / 10  Mod/sev: 5.9 / 20 / 34.8 / 75 | - | Unemployment - CD:26.8%, UC: 26.6% |
| Christiansen 2019^35^ | CD: 5.6 / 11.7 / 16 / 22.6  UC: 6.5 / 9.6 / 14.6 / 16.4 | - | 47.8% patients reported sick leave within last 3 yrs  18.1% reported more than one yr unemployment within last 3 yrs  Unemployment - CD: 6.7%, UC: 3.8%  12.5% reported more than 3-12 months of unemployment within last 3 yrs  30.6% overall unemployment within last 3 yrs |
| Pillai 2019^36^ | - | Indirect cost for absenteeism -  CD: 1339 ± 8576.5, UC: 707 ± 3124.4 | Unemployment: 11% |
| LeBerre 2019^37^ | Total (n=1336): 10.9 / 34.3 / 36 / 46.9  Mild: 5.8 / 25.4 / 26.1 / 35.1  Mod: 9.8 / 32.6 / 34.4 / 45.5  Sev: 15.2 / 42.7 / 44.4 / 56 | - | 5% disability due to IBD  Unemployment: 7% |
| Gonczi 2019^38^ | Total: 6.8 / 15.9 / 22.7 / -  CD: 6.1 / 15.9 / 22 / -  UC: 9.5 / 15.8 / 25.3 / - | - | - |
| Everhov 2019^39^ | - | - | Mean sick days - Before diagnosis: 14 days, During diagnosis: 31 days, After diagnosis: 18 days  %patients reporting sick days - 22% during year before diagnosis, 34% during year of diagnosis  9-11% disability pensions  Unemployment: 9.9% |
| Everhov 2018^40^ | - | - | Mean 19 sick days  19% patients reporting sick days  15% disability pensions  62 lost workdays total (14 sick days and 44 disability pension days)  32% of patients lost workdays  11% of patients had total work loss (365 days)  21% of patients had partial work loss (1-364 days) |
| Spekhorst 2017^41^ | - | - | %patients reporting sick days - CD: 4%, UC: 3%  % disability due to IBD - CD: 29%, UC: 19% |
| Kamat 2017^42^ | - | Absenteeism -  CD: $47.8 USD  UC: $40 USD | Mean sick days - CD: 14-39, UC: 12-34  Lost workdays - CD: 9, UC: 8  CD - Duration of unemployment: 97 days  UC - Duration of unemployment: 21 days  Unemployment - CD: 12%, UC: 8.5% |
| Williet 2017^43^ | Total (MEDIAN): 0 / 17.5 / 20 / 30  CD (MEDIAN): 0 / 20 / 20 / 30  UC (MEDIAN): 0 / 10 / 17.5 / 30 | - | Severe absenteeism reported in 6.4%  Severe presenteeism reported in 18.8%  Severe work productivity loss reported in 20.3%  Severe activity impairment reported in 34.9%  Unemployment - Total: 11.9, CD: 13.3, UC: 9.4 |
| Holko 2016^44^ | - | Total IBD - Mean absenteeism cost: €186.66 ± 352.45, Presenteeism: €235.09 ± 250.40/patient/mo  Active -Mean absenteeism cost: €157.43 ± 336.61, Presenteeism: €169.51 ± 249.12  Productivity loss at unpaid work: €77.89 ± 143.44/patient/mo  Remission - Mean absenteeism cost: €59.61 ± 210.42, Presenteeism: €102.10 ± 183.52  Productivity loss at unpaid work: €18.88 ± 64.40/patient/mo  Total indirect costs: €462.47/patient/mo | %disability pensions - Total (n= 200): 10.5%, Active (n= 105): 15.2%, Remission (n= 93): 4.3% |
| DeBoer 2016^45^ | - | - | 25% (N= 123) patients reporting sick days  23% (N= 123) disability pensions  Unemployment: 0.5% |
| Aldeguer 2016^46^ | - | Total indirect costs: 399.32 ± 621.47  Sick leave costs: 311.11 ± 610.09  Absenteeism: 88.21 ± 70.19 | Mean 26.17 ± 37.43 sick days  33.50% patients reporting sick days |
| Vester-Andersen 2015^47^ | - | - | CD: 52.3% reported sick leave >4 wks  UC: 43.3% reported sick leave >4 wks  5.8% disability pensions  Unemployment - CD: 7.9%, UC: 11.3% |
| Zand 2015^48^ | Total: 21.2 / 62.9 / - / 63.6  CD (n=140): 2- / 61.4 / - / 66.4  UC (n=143): 22.4 / 64.3 / - / 66.4  Active (n=58): 46.6 / 94.8 / - / 98.6  Remission (n=225): 14.4 / 54.7 / - / 62.7 | Active - Total indirect costs: $1133/wk, Presenteeism: $695.03/wk, Absenteeism: $437.9 per week  Remission - Total indirect costs: $370.13/wk, Presenteeism: $277.60/wk, Absenteeism: 4.5% of total weekly compensation | 3.30% disability pensions  Unemployment: 35.6% |
| Cohen 2015^49^ | - | Total: $4125 USD  Mod/Sev UC: $5666 USD | Mean sick days - Total: 10.9%, Mod/sev UC: 16.7%  %patients reporting sick days - Total: 98.7%, Mod/sev UC: 98.2%  Number of workdays missed - Total: 16.4 days, Mod/sev UC: 23.8 day |
| vanderHave 2015^50^ | - | - | Total self-reported work disability: CD: 50%, UC: 35% |
| Mandel 2014^51^ | CD: 8.5 / 27.6 / 28.8 / 33.9  UC: 11.7 / 24.5 / 27.6 / 31 | Total: 1450  CD: 1545  UC: 1310 | 32.3% disabilty pensions  Mean working hours loss - Total:1.39 hrs/week, CD:1.28 hrs, UC: 1.56hrs |
| Lonnfors 2014^52^ | - | - | 29% patients had been absent 1–10 days, 16% 11–25 days, 25% >25 days in 1 yr |
| vanderValk 2014^53^ | - | - | Self-reported full disability - CD (N= 728): 18.3%, UC (N= 605): 9.5%  Partial disability - CD (N= 728): 8.8%, UC (N= 605): 5.4% |
| Gibson 2014^54^ | Total (n=175): 19 / 22.4 / 28 / 30.9  Remission (n=94): 8.5 / 16.7 / 20.8 / 18.1  Mild (n=29): 2.5 / 17.8 / 19.6 / 34.5  Mod/sev (n=52): 43.4 / 36.9 / 48.2 / 52.2 | - | Unemployment: 6.3% |
| Siebert 2013^55^ | - | - | Permanent disability - CD: 9.7%, UC: 3.1%  Temporary disability - CD: 19.3%, UC: 21.3%  Temporary disability days - CD: 3.7±14 days, UC:5.6±18.3 days |
| Gunnarsson 2013^56^ | - | $782.77/year USD | 71.5% patients reporting sick days  Mean 13.38 days/year missed |
| Hoivik 2013^57^ | - | - | %dsiability due to IBD: UC: 18.5%, CD: 19.4%, Total IBD: 18.8%  %disability pensions: UC: 9.4%, CD: 15.6% |
| Ramos 2015^58^ | - | - | 27.4 ± 71 mean sick days  31.8% disability due to IBD  5.4% disability pensions  Unemployment: 5.5% |
| Vaizey 2014^59^ | Total (n=173): 11.9 / 21.6 / 25.3 / 26.1  Remission (n=100): 1.8 / 12.9 / 14.4 / 13  Active disease (n=73): 24.6 / 34.1 / 40.8 / 42.7 | - | 21.6% impairment due to IBD  %disability pensions - All patients (N=173): 6.9%, Remission (N=97): 8.2%, Active disease (N=73): 5.5%, Mild (N=31): 6.5%, Mod/sev (N=42): 4.8%  Unemployment: 5.8% |
| Viazis 2013^60^ | - | - | Days off of work due to IBD during the last yr - 1-5 days: 24%, 6-10 days: 11%, 11-15 days: 5%, 16-20 days: 14%, >20 days: 3% |
| Benedini 2012^61^ | - | €1923.6/patient | - |
| Zhou 2010^62^ | - | - | Unemployment - CD: 25, UC: 9.6 |
| Gibson 2008^63^ | - | CD: $236.18 USD  UC: $631.72 USD | - |
| Stark 2006^64^ | - | For average 4 weeks due to productivity loss -  CD: €218, UC: €224  Longterm productivity loss -  CD: €700, UC: €236 | %disability due to IBD - CD: 19%, UC: 7%  %disability pensions - CD: 19%, UC: 16%  Unemployment - CD: 3%, UC: 3% |
| Bernklev 2006^65^ | - | - | % patients reporting sick days - CD: 53%, UC: 47%  % disability due to IBD: CD:14.9%, UC:5.4%  % disability pensions: CD:14.9%, UC:5.4%  Unemployment - CD: 8.7, UC: 13.2 |
| Boonen 2002^66^ | - | - | Mean sick days - All IBD: 13.2 ± 36.5, CD: 16.7 ± 42.4, UC: 10.1 ± 28.7  Unemployment - CD: 2.1%, UC: 1.1% |
| Bernstein 2001^67^ | - | - | 1.3% disability due to IBD (Study A, all IBD) - CD: 4%, UC: 1.3%  Study B labour force participation: 71.4%  Unemployment: Study A:  All IBD (M, N=1074): 7.1%  All IBD (F, N=1402): 7.3%  CD (M, N=495): 9.2%  CD (F, N=736): 8.7%  UC (M, N=579): 6.0%  UC (F, N=666): 6.3% |
| Sorensen 1987^68^ | - | - | Unemployment: 6% |
| Sikirica 2022^69^ | Colonic: 8.1 (n=134) / 33.6 (n=33.6) / 35.5 (n=127) / -  Ileal: 12.8 (n=141) / 30.7 (n=134) / 32 (n=125) / -  Ileocolonic: 17.4 (n=202) / 33.1 (n=208) / 38.8 (n=188) / - | - | - |
| Tiankanon 2021^70^ | - / - / 17.8 ± 25.3 / - | - | - |
| Humberto 2021^71^ | - | - | Mean absences from work: 4.4 days |
| Wong 2020^72^ | Mild: 16.55 ± 25.20 (n=252) / 34.72 ± 34.94 (n=250) / 38.44 ± 37.25 (n=248) / 36.47 ± 32.78 (n=464)  Mod/sev: 28.19 ± 26.58 (140) / 58.90 ± 31.82 (n=145) / 65.31 ± 33.59 (n=140) / 56.95 ± 31.30 (n=233) | - | Unemployment - Mild: 40.6, Moderate/severe: 37.3 |
| Armuzzi 2019^73^ | 5.5 / 22.9 / 24.5 / 55.1 | - | Unemployment: 23% |
| Limdi 2019^74^ | Arm 1 (n=602): 11 ± 25 / 24 ± 21 / 28 ± 24 / 27 ± 24  Arm 2 (n=368): 1 ± 19 / 24 ± 18 / 20 ± 21 / 18 ± 20 | - | - |
| Carels 2019^75^ | - / - / 15 / 26 | - | - |
| Raimundo 2018^76^ | Mild (n=620): 12.6 / 27.36 / 32.53 / 34.42  Mod (n=307): 23.8 / 47.33 / 54.54 / 53.45  Sev (n=93): 23.99 / 44.65 / 51.74 / 55.38 | (Annual USD)  Mild (n=620): $15769  Mod (N=307): $27292  Sev (N=93): $28818 | - |
| Armuzzi 2018^77^ | Active disease (n=349): - / - / 54.4 / 57.6  Remission (n=550): - / - / 19.4 / 18.9  Deep remission (n=138): - / - / 13.8 / 15.7 | - | - |
| Sebastian 2018^78^ | - / - / - / 26 | - | - |
| Aiello 2018^79^ | **11.6 / - / - / - | - | Unemployment: 50.6% |
| DeLima 2018^80^ | - | - | 8% disability pensions  8% with temporary benefits  Unemployment: 45% |
| Hellstrom 2017^81^ | - | (Annual Swedish SEK)  CD: 86440, UC: 50925 | Mean sick days - CD: 16wks, UC: 9wks |
| Ghosh 2017^82^ | Remission: 5.6 ± 19.8 (n=145) / 12.9 ± 22.8 (n=146) / 15.4 ± 25.5 (n=138) / 13.1 ± 22.1 (n=226)  Mild: 9.7 ± 24.2 (n=407) / 21.1 ± 26.6 (n=417) / 24.7 ± 29.8 (n=395) / 25.1 ± 26.2 (n=663)  Mod: 20.4 ± 33.9 (n=383) / 33.7 ± 26.9 (n=374) / 39.7 ± 31.5 (n=353) / 42.6 ± 28.1 (n=659)  Sev: 38.9 ± 42.2 (n=115) / 52.8 ± 33.1 (n=105) / 62.1 ± 33.1 (n=99) / 58.9 ± 28.2 (n=229) | - | 10.1% patients reporting sick days  Unemployment - Remission (n=230): 16.7, Mild (n=672): 18.4, Mod (n=668): 21.6, Sev (n=234): 39.4 |
| Ganz 2016^83^ | - | USD $1249 ± 4720.3/person/yr | 4.5 ± 16.0 lost workdays/person/yr  Unemployment: 18% |
| KatzAvitan 2016^84^ | CD: 7.6 / 26 / - / -  UC: 9.2 / 19 / - / - | - |  |
| Camacho 2016^85^ | - / - / 42 / 52 | - | 1.63 mean working hours loss  Unemployment: 22% |
| Schwartz 2016^86^ | **Remission: 1.3 ± 3.4 hrs / - / - / -  Mild: 2.9 ± 5.5 hrs / - / - / -  Mod: 8.0 ± 11.7 hrs / - / - / -  Sev: 10.7 ± 10.3 hrs / - / - / - | - | - |
| VanAssche 2015^87^ | 12.3 ± 27.3 / 20.5 ± 25.9 / 26.5 ± 32.2 / 17.1 ± 25.5 (controlled), 27.4 ± 28.6 (uncontrolled) | - | Unemployment: 40.7% |
| Huascar 2015^88^ | - | - | %disability due to IBD - Mod: 26%, Sev:6% |
| Carpio 2015^89^ | - | - | 55% reporting sick leave |
| Geccherle 2015^90^ | - / - / 22.15 / - | - | - |
| Piercy 2015^91^ | CD: 16.1 ± 30.7 (n=305) / 31.5 ± 23.4 (n=284) / 34.8 ± 26.0 (n=275) / 38.9 ± 26.5 (n=577)  UC: 14.6 ± 29.3 (n=232) / 31.9 ± 22.4 (n=219) / 35.1 ± 24.1 (n=211) / 40.0 ± 26.3 (n=487) | - | - |
| Miller 2014^92^ | - | - | 70% of patients reporting sick days (within a year)  <10% disability pensions |
| Wladysiuk 2014^93^ | 18 / 27 / 36 / - | - | - |
| Zand 2014^94^ | - / - / 21 / 62 | - | Unemployment: 36% |
| Kroeker 2012^95^ | - | - | WHO HPQ Absenteeism - CD (n=83): 14.1 ± 4.00, UC (n=55): 18.0 ± 5.77  Mean full sick days - CD: 0.7 ± 0.16, UC: 0.65 ± 0.16  Mean part sick days - CD: 0.4 ± 0.14, UC: 0.6 ± 0.17  % disability due to IBD - CD: 2.4%, UC: 1.4%  Other missed days - CD: 1.3 ± 0.28, UC: 1.9 ± 0.51  Unemployment - CD: 10.9, UC: 6.8 |
| Cohen 2012^96^ | - | $4047 USD (annual) | Mean medically-related absenseeism days: UC: 10.2, Mod/sev UC: 14.2  Mean disability days: UC: 8.8, Mod/sev UC: 15.0 |
| Wilson 2012^97^ | - | - | 44% lost job due to illness |
| Naim 2011^98^ | 9.97 / 16.3 / 20 / 43.2 | - | 13% disability due to IBD |
| Gomollon 2011^99^ | - / - / 26.9 / 35 | - | 34%: at least 1 hour or work loss per week due to CD  3.54: mean number of work hour loss due to CD in previous 7 days |
| Kane 2009^100^ | - | - | Mean working hours loss - 9.9 (full-time), 8.9 (part-time)  Unemployment: 41% |
| Procaccini 2007^101^ | - | - | 20.4% disability due to IBD  Unemployment: 32.3% |
| Yan 2020^102^ | - | - | % patients reporting sick leave - CD: 13.2%, UC: 4.7%, Total: 9.7%  Lost workdays - CD: 71.1 ± 118.2, UC: 31.7 ± 84.3  Unemployment - CD: 6.9, UC: 3, Total: 5.3 |
| Ueno 2017^103^ | - | - | 64.5% patients reporting sick days |
| VanDerValk 2014^104^ | - | CD: Mean €325.73/patient/3mo  UC: Mean €395.21/patient/3mo | Mean sick days - Paid work - CD: 2.5, UC: 2.5, Unpaid work - CD: 5.4, UC: 7.8  % patients reporting sick days - Paid work - CD: 18.3%, UC: 13.4%, Unpaid work - CD: 4.7%, UC: 3.0%  Fully work disabled - CD: 17.5%, UC: 9.8%  Partial work disabled - CD: 8.3%, UC: 4.9% |
| Hendrikson 1980^105^ | - | - | 69% patients reporting sick days  2% disability due to IBD  84% full work-capacity  Unemployment: 2% |
| Binder 1985^106^ | - | - | 12-20% disability due to IBD at 5 years of disease |
| Nurmi 2013^107^ | - | - | % patients reporting sick days - Total: 26.7%, CD: 30.6%, UC: 24.9% |
| Ghosh & Mitchell 2007^108^ | - | - |  |
| Stjernman 2011^109^ | - | - | Reduced capacity to work - CD: 27%  % disability due to IBD - CD: 16%  Labour force participation (N=327) - CD: 65.7%  Unemployment - CD: 4% |
| Longobardi 2003^110^ | - | $3.6B USD in 1998/1999 OR $5228 USD/person | Labour force participation - w sx: (N=73): 69%; w/o sx (N=67): 82%  Non-participation - w sx (N=33): 31%; w/o sx (N=15): 18% |
| Longobardi 2003^111^ | - | $104.2 M CAD in 1998 OR $868 CAD/person | Labour force participation (N=133): 71%  Non-participation (N=46): 29% |
| Mayberry 1992^112^ | - | - | 6.8% lost job due to illness |
| Gazzard 1978^113^ | - | - | 5% receiving temporary benefits |
| Juan 2003^114^ | - | €4704/patient/yr | 36% patients reporting sick days  % disability due to IBD - Total: 9%, Partial: 6%  3.3 ± 2.4 mean working hours loss  Unemployment: 37% |
| Ananthakrishnan 2008^115^ | - | - | 5% disability pensions |
| Blomqvist 1997^116^ | - | Sickness leave: 27.6M USD  Early retirement: 30.8M USD  Total: 58.4M USD in 1994 | Mean sick days - CD: 44, UC: 58, Total: 45 |
| Mesterton 2009^117^ | - | Estimated 4-week cost due to productivity loss: €465 | - |

IBD: Inflammatory Bowel Disease, CD: Crohn’s disease, UC: Ulcerative Colitis, IC: Indeterminate Colitis. M: Male, F: Female. Mod: moderate, Sev: severe.

**WPAI defined as mean time

**Supplementary Table S2. Interventional Studies - Baseline Characteristics and Study Outcomes**

| Study | Type of intervention | Sample Size | %Male, Female | Mean age ± SD | %Employment | WPAI reported? | WPAI (% ± SD)  Absenteeism / Presenteeism / TWPI / OAI  **PRE-Intervention** | WPAI (% ± SD)  Absenteeism / Presenteeism / TWPI / OAI  **POST-Intervention** | Miscellaneous Outcomes |
| --- | --- | --- | --- | --- | --- | --- | --- | --- | --- |
| Reilly 2008^118^ | Medical (Certolizumab) | CD=622 | M:43.5, F:56.5 | 37.4 | 61 | ✓ | 18.3 ± 29.3 (n=351) / 40.5 ± 25.2 (n=359) / 45.9 ± 26.5 (n=326) / 52 ± 25.2 (n=641) | - | - |
| Yarlas 2015^119^ | Medical (Mesalamine) | UC=343 (Active=132, Maintenance=206) | Active - M:43.9, F:56.1  Quiescent - M:49.5, F:51.5 | Active: 43.4  Quiescent: 46.9 | Active: 63.6  Quiescent: 72.8 | ✓ | Acute sample baseline (n=132): 8.8 ± 21.1 / 27.5 ± 26.3 / 30 ± 29.2 / 35.5 ± 31.2  Maintenance baseline (n=206): 6.8 ± 18.2 / 25.2 ± 24.5 / 27.4 ± 26.9 / 32.4 ± 30.14 | Acute phase sample at 8 wks*: 2.8 ± 12.88 (n=70) / 12.9 ± 21.51 (n=69) / 13.6 ± 22.49 (n=69)/ 18.2 ± 25.778 (n=103) | - |
| Willian 2018^120^ | Medical (Mesalamine) | UC=1176 (Acute=717, Maintenance=459) | Acute phase: M:57, F:43  Maintenance: M:56.4, F:43.6 | Acute: 42.9 ± 14.0  Maintenance: 42.7 ± 14.2 | - | ✓ | Acute Phase: 13.0 ± 25.8 / 35.9 ± 26.5 / 42.0 ± 30.5 / 41.5 ± 26.9  Maintenance: 12.1 ± 25.7 / 32.6 ± 26.3 / 38.9 ± 30.95 / 38.9 ± 27 | Acute Phase at 8wks*: 4.5 ± 0.8 / 16.7 ± 1.19 / 19.8 ± 1.39 / 19.3 ± 1.02  Maintenance at 12mo*: 3.8 ± 14.99 / 13.4 ± 23.39 / 15.7 ± 27.3/ 15.4 ± 23.25 | - |
| Yarlas 2014^121^ | Medical (MMX mesalazine) | varies based on result | - | - | - | ✓ | Acute efficacy population: 10.8 ± 20.9 (n=198) / 35.2 ± 24.5 (n=199) / 40.3 ± 27.9 (n=190) / 41.3 ± 25.1 (n=384)  Maintenance efficacy population: 11.4 ± 21.4 (n=74) / 39.9 ± 23.9 (n=71) / 38.7 ± 27.0 (n=69) / 39.8 ± 23.8 (n=177) | Acute efficacy population -  *Wk3: 4.6 ± 16.0 (n=198) / 19.1 ± 19.4 (n=199) / 22.3 ± 23.3 (n=190) / 24.9 ± 22.3 (n=384)  *Wk8: 3.6 ± 13.5 (n=198) / 14.1 ± 19.2 (n=199) / 16.4 ± 23.0 (n=190) / 16.2 ± 19.7 (n=384)  Maintenance efficacy population -  *Wk3: 3.5 ± 12.2 (n=74) / 14.7 ± 13.5 (n=71) / 16.9 ± 17.7 (n=69) / 20.3 ± 18.0 (n=177)  *Wk8: 2.4 ± 12.0 (n=74) / 7.3 ± 12.2 (n=71) / 9.6 ± 17.2 (n=69) / 11.4 ± 14.8 (n=177)  *12mo: 2.6 ± 12.2 (n=74) / 7.2 ± 15.6 (n=71) / 9.3 ± 19.9 (n=69) / 10.6 ± 15.4 (n=177) | - |
| West 2022^122^ | Medical (Mesalazine) | UC=151 | M:51.7, F:48.3 | - | Baseline: 72.8  12mo: 69.7 | ✓ | **5.4 ± 11.4 / 3.6 ± 3.4 / - / 4.0 ± 3.4 | **12mo: 2.7 ± 8.1 / 2.0 ± 3.2* / - / 2.4 ± 3.3* | - |
| Bessissow 2022^123^ | Medical (Adalimumab) | UC=94 | M:59.6, F:40.4 | 42.5± 15.3 | 67 | ✓ | 18.9 ± 31.1 / 39.5 ± 28.5 / 44.2 ±30.1 / 46 ± 31.9 | Change at 8 wks: -6.7 ± 30.9 (n=48) / -14.8  ± 33.1 (n=46) / -16.2 ± 30.2 (n=42) / -16.9 ± 29.8 (n=83)  Change at 52 wks: -9.4 ± 35 (n=37) / -14.5 ± 35.8 (n=38) / -14.5 ± 34.4 (n=35) / -16.7 ± 33.6 (n=64) | 2% ± 2.1% disability due to IBD  Unemployment: 6% |
| Loftus 2019^124^ | Medical (Adalimumab) | CD=2057 | M:41.7, F:58.3 | 37.1 ± 12.7 | - | ✓ | 20.18 / 39.53 / 47.68 / 50.45 | 1yr^⤉^: 8.01 (n=314) / 20.68 (n=338) / 24.3 (n=310) / 28.42 (n=664) | - |
| Travis 2017^125^ | Medical (Adalimumab) | UC=463 | M:55.3, F:44.7 | 41.8 ± 13.8 | - | ✓ | 20.4 ± 30.1 (n=223) / 50.2 ± 24.8 (n=229) / 58.5 ± 26.7 (n=221) / 59.0 ± 23.5 (n=446) | Change at 26 wks*^⤉^: -11.4 (n= 223) / -24.5 (n=229) / -29.2 (n=221) / -27.2 (n=446) | - |
| Louis 2013^126^ | Medical (Adalimumab) | CD=945 | M:39.9, F:60.1 | 35.3 ± 11.3 | 57.4 | ✓ | Baseline: 23.1 ± 34.4 (n=468) / 45.4 ± 27.2 (n=484) / 51.9 ± 29 (n=442) / 56.6 ± 25.8 (n=907) | Change at 4 wks*^⤉^: -9.6 ± 30.0 (n=353) / -17.3 ± 27.1 (n=395) / -18.4 ± 30.0 (n=327) / -21.3 ± 27.0 (n=856)  Change at 20 wks*^⤉^: -9.8 ± 31.7 (n=328) / -20 ± 30.9 (n=360) / -21.4 ± 33.6 (n=302) / -25.9 ± 29.7 (n=747) | Indirect costs - All adalimumab at 20wks - Absenteeism: €1180, Presenteeism: €2408, TWPI: €2577 |
| Lichtiger 2010^127^ | Medical (Adalimumab) | CD=673 | M:40, F:59.1 | 40.8 | 62.9 | ✓ | All adalimumab (n=673): 14.8 ± 25.1 / 44.3 ± 27 / 49.4 ± 29.3 / 57.9 ± 26.9 | - | - |
| Panaccione 2011^128^ | Medical (Adalimumab) | CD=304 | M:43.1, F:56.9 | 37 ± 12 | Baseline: 64.1  Final visit: 69 | ✓ | Baseline (n=304): 16 ± 28 / 50 ± 25 / 57 ± 27 / 63 ± 24 | *Wk4: - / - / 33 (n=182) / 39 (n=297)  *Wk8: - / - / 33 (n=204) / 39 (n=297)  *Wk12: - / - / 29 (n=214) / 35 (n=303)  *Wk24: - / - / 29 (n=214) / 33 (n=303) | - |
| Toruner 2022^129^ | Medical (Infliximab or Adalimumab) | CD=106 | M:64.2, F:35.8 | 36.8 ± 10.9 | - | ✓ | 38.3 ± 39.0 (n=67) / 39.8 ± 31.7 (n=58) / 24.2 ± 21.2 (n=57) / 47.0 ± 32.0 (n=105) | 1mo: 26.8 ± 37.4 / 31.9 ± 30.4 / 22.7 ± 22.2 / 33.8 ± 29.1  12mo*: 14.2 ± 27.0 (n=33) / 21.8 ± 19.3 (n=40) / 15.7 ± 16.4 (n=33) / 30.0 ± 27.0 (n=62) | - |
| Ding 2021^130^ | Medical (Ustekinumab vs. Adalimumab) | CD=386 | - | - | - | ✓ | Ustekinumab: 19.8 ± 30.2  (n=118) / 52. 9 ± 26.4 (n=116) / 59.6 ± 27.8 (n=113) / 57.3 ± 24.2 (n=188)  Adalimumab: 20.7 ± 30.1 (n=136) / 48.0 ± 25.7 (n=132) / 54.0 ± 27.6 (n=130) / 51.3 ± 24.6 (n=192) | Change at Wk52 -  Ustekinumab: -13.8 ± 30.4 (n=85) / -32.2 ± 30.8 (n=97) / -34.6 ± 35.0 (n=80) / -30.1 ± 29.0 (n=172)  Adalimumab: -9.9 ± 25.0 (n=90) / -24.3 ± 26.1 (n=94) / -26.8 ± 28.0 (n=86) / -26.1 ± 26.7 (n=167) | Indirect costs - (Annual USD)  Ustekinumab - Baseline: $36029, Post-treatment: $14740  Adalimumab - Baseline: $34024, Post-treatment: $18493 |
| Dignass 2019^131^ | Medical (Adalimumab, Golimumab, Infliximab, Vedolizumab) | UC=304 | - | - | - | - | - | - | 39.2 mean sick days  47.7% disabiity due to IBD  Mean sick benefits: €832 ± 3664 |
| Feagan 2005^132^ | Medical (Infliximab) | CD=573 | M:41.7, F:58.3 | 37 ± 12 | 48 | - | - | - | 25% disability pensions  Unemployment: 39% |
| Teich 2021^133^ | Medical (Infliximab) | CD=294 | M:47.3, F:52.7 | - | 50 | ✓ | - / - / 41.2 (n=144) / 48.6 (n=288) | Change at 3 mo*: -12.6 (n=91) / -17.1 (n=249) / -12.6 (n=91) / -17.1 (n=249) | Number of workdays missed - Baseline: 55 days/3mo, 24mo: 24 days/3mo |
| Perampaladas 2013^134^ | Medical (Infliximab) | Total=846 (CD=609, UC=237) | M:51, F:49 | 39 ± 20 | 72 (full- and part-time) | - | - | - | % patients reporting missing >5 days of work/school - Before therapy: 33%, After therapy: 7%  % patients receiving disability insurance - Before therapy: 33%, After therapy: 9%  Unemployment - 10% |
| Gatopoulou 2021^135^ | Medical (Golimumab) | UC Baseline total=81  UC 12mo total=53 | M:54.3, F:45.7 | - | Baseline: 45.6  12mo: 27.1 | ✓ | 27.4 ± 36.3 / 40.6 ± 30.5 / 24.7 ± 21.0 / 43.4 ± 28.8 | 6mo: 9.5 ± 26.2 / 20.3 ± 28.3 / 12.1 ± 16.3 / 23.9 ± 29.2  12mo: 9.1 ± 23.9 / 11.8 ± 27.0 / 5.9 ± 13.3* / 15.3 ± 24.9* | - |
| Teich 2020^136^ | Medical (Golimumab) | UC=287 | M:47.2, F:52.8 | 40.8 ± 13.9 | 74.8 | ✓ | 27.6 ± 37.7 (n=180) / 45.3 ± 26.0 (n=145) / 49.7 ± 27.7 (n=143) / 52.8 ± 26.9 (n=205) | Change at 3 mo*: -13.8 ± 38.8 (n=144) / -14.9 ± 28.8 (n=128) / -17.3 ± 32.2 (n=127) / -14.4 ± 28.5 (n=157)  Change at 12 mo*: -19.9 ± 42.0 (n=110) / -22.5 ± 29.2 (n=75) / -23.7 ± 30.8 (n= 71) / -27.5 ± 29.3 (n= 117) | Unemployment: 16.3% |
| Feagan 2010^137^ | Medical (Certolizumab) | CD=428 (Placebo=210, Treatment=215) | Placebo - M:51.9, F:48.1  Treatment - M:42.8, F:57.2 | Placebo: 37.6  Treatment: 37.5 | Placebo: 67.6  Treatment: 64.2 | ✓ | Placebo: 17.9 ± 27.8 (n=124) / 40.0 ± 22.5 (n=125) / 45.2 ± 25.2 (n=116) / 51.3 ± 23.6 (n=205)  Certolizumab: 21.7 ± 30.3 (n=119) / 43.3 ± 24.5 (n=116) / 49.5 ± 25.7 (n=109) / 51.2 ± 24.8 (n=208) | Change at 6 wks:  Certolizumab*^⤉^: -12.8 (n=202) / -19.9 (n=210) / -25.6 (n=187) / -25.0 (n=409)  Change at 26 wks:  Placebo: -2.3  ± 34.5 (n=100) / -2.5  ± 34.5  (n=101) / -6.7  ± 37.2 (n=91) / -9.6  ± 32.9* (n=193)  Certolizumab*: -12.2  ± 29.7 (n=102) / -17.9  ± 27.2 (n=99) / -21.0  ± 29.3 (n=91) / -18.7  ± 28.9 (n=199) | - |
| Carlucci 2020^138^ | Medical (Ustekinumab) | UC=206 | - | - | - | - | - | - | Indirect costs (Annual USD)  Absenteeism (UST 90 Q8W) - Baseline: $9614, Wk52: $4727,  Absenteeism (UST 90 Q8W/Q12W) - Baseline: $10152, Wk52: $4112  Presenteeism (UST 90 Q8W) - Baseline: $21083, Wk52: $10297  Presenteeism (UST 90 Q8W/Q12W) - Baseline: $20564, Wk52: $9290.  Total WPL (UST 90 Q8W) - Baseline: $30697, Wk52: $15024.  Total WPL (UST 90 Q8W/Q12W) - Baseline: $30716, Wk52: $13402. |
| D'Haens 2020^139^ | Medical (Vedolizumab) | CD=409 | Placebo - M:49.3, F:50.7  Vedolizumab - M:57.1, F:42.9 | Placebo: 36.1 ± 12.9  Vedolizumab: 38.2 ± 13.9 | - | ✓ | Placebo: - / - / 58.6 (n=72) / -  Vedolizumab: - / - / 59.6 (n=136) / - | ^⤉^Placebo at Wk6: - / - / 31.3 (n=77) / -, Wk30: - / - / 39.3 (n=71) / -,  Wk52: - / - / 33.0 (n=46) / -  ^⤉^Vedolizumab at Wk6: - / - / 30.7 (n=148) / -, Wk30: - / - / 34.6 ( n=146) / -, Wk52: - / - / 25.7 (n=111) / - | - |
| Danese 2019^140^ | Medical (Anti-TNF) | CD=56 | M:54, F:46 | 39.6 | - | ✓ | Overall: 22.8 ± 37.2 (n=23) / 38.3 ± 29.2 (n=23) / 47.7 ± 31.2 (n=22) / 51.1 ± 27.8 (n=53)  Remission: 17.2 ± 32.9 (n=9) / 30.0 ± 23.5 (n=9) / 45.0 ± 30.0 (n=9) / 50.6 ± 27.2 (n=16)  No remission: 26.5 ± 40.4 (n=14) / 43.6 ± 32.0 (n=14) / 49.6 ± 33.1 (n=13) / 51.4 ± 28.4 (n=37) | 14wks - 7.0 ± 20.8 (n=28) / 23.8 ± 23.5 (n=29)^⤉^ / 28.0 ± 26.2 (n=28)^⤉^ / 33.5 ± 23.6 (n=51)^⤉^  52wks - 6.7 ± 13.0 (n=35) / 19.7 ± 25.1 (n=36)^⤉^ / 21.5 ± 24.6 (n=35)^⤉^ / 28.4 ± 27.2 (n=56) | - |
| Ghosh 2018^141^ | Medical (Upadacitinib) | UC=2-50 | M:60, F:40 | 42.3 | - | ✓ | Baseline: - / - / 51 / 52 | Change at 8wks -  Placebo: - / - / -7.7 (n=44) / -0.8 (n=27)  UPA 7.5mg QD: - / - / -14.8 (n=44) / -10.5 (n=27)  *UPA 15mg QD: - / - / -17.1 (n=48) / - 22.6 (n=30)  *UPA 30mg QD: - / - / -24.2 (n=48) / -19.1 (n=33)  *UPA 45mg QD: - / - / -27.2 (n=54) / -23.0 (n=39) | - |
| Reinisch 2007^142^ | Medical (ASAs, steroids, or immunomodulators) | UC=728 | M:60, F:40 | 40.9 ± 13.8 | 64.6 | - | - | - | 19.8% disability due to IBD  Baseline -  Remission (n= 208) - Hours pr wk actually worked: 22.8, Productivity: 68.5 (n=68), Hours per wk fully productive: 13.8 (n=106)  Active (n= 290) - Hours per wk actually worked: 21.1, Productivity: 67.6 (n=63), Hours per wk fully productive: 12.9 (n=119)  Week 30 -  Remission (n= 208) - Hours pr wk actually worked: 18.4, Productivity: 44.9 (n=68), Hours per wk fully productive: 4.1 (n=106)  Active (n= 290) - Hours per wk actually worked: 20.9, Productivity: 54.3 (n=63), Hours per wk fully productive: 9.8 (n=119)  Unemployment - 35.4% |
| Cross 2022^143^ | Medical (5-ASA, Biologic/JAKi) | UC=605 | M:46, F:54 | 47.3 ± 17.0 | 66.7 | ✓ | Total: 5.3 ± 18.2 (n=368) / 15.5 ± 24.4 (n=399)/ 17.4 ± 26.3 (n=367) / 19.8 ± 27.0 (n=603)  5-ASA: 2.8 ± 12.0 (n=162) / 13.7 ± 22.6 (n=180) / 15.1 ± 23.6 (n=162) / 17.5 ± 25.6 (n=290)  BIO/JAKi: 7.3 ± 21.8 (n=206) / 17.0 ± 25.6 (n=219) / 19.2 ± 28.2 (n=205) / 22.0 ± 28.0 (n=313) | - | - |
| Neovius 2013^144^ | Surgical (Colectomy) | UC=19714 | M:54, F:46 | 44 ± 12 | - | - | - | - | Mean sick days - Total UC (n=19,557): 65 days, No colectomy (n= 16,514): 60, With colectomy (n=3043): 92  21% patients reporting sick days  15% disability pensions |
| Damgaard 1995^145^ | Surgical (Ileal J-pouch-anal anastomosis) | UC=49 | M:53, F:47 | - | Full-time: 33  Half-time: 12 | - | - | - | 31% resumed work in the period with diverting ileostomy  Reduced capacity to work preop: 88%  Reduced capacity to work postop: 6%  Unemployment: 10% |
| Wyke 1988^146^ | Surgical (Colectomy + IRA, PCC & ileostomy, segmental resection, and ileostomy) | Total=144 (CD=83, UC=61) | 48 ± 12 | 65.3 | Full-time - Baseline: 83, 6yr: 70  Part-time - Baseline: 24, 6yr: 19 | - | - |  | No change 44% (53/120)  Had to change 10% (12/120)  Modified work/hours 13% (15/120)  Retrained 2% (2/120)  Premature retirement 4% (5/120)  Lighter work 3% (4/120)  Change not due to health 24% (29/120)  Unemployed - Baseline: 3%, 6yr: 7%  Sickness absence was attributed to bowel problems in 44%, general malaise 44%, and miscellaneous 12% |

IBD: Inflammatory Bowel Disease, CD: Crohn’s disease, UC: Ulcerative Colitis, IC: Indeterminate Colitis. M: Male, F: Female. Wk: week, Mon: month. UST: Ustekinumab, WPL: Work productivity loss,

*Statistically significant difference

**WPAI defined as mean time

⤉Clinically meaningful improvement

**Supplementary Table S3: Risk of bias of cross-sectional studies as per the Appraisal tool for cross-sectional studies (AXIS) tool**

| **Cross-sectional studies** | Study Aims | Study Design | Sample size | Target population | Representative sample? | Selection process | Measures to address non-responders? | Risk factors & outcomes | Study measurements | Statistical significance | Statistical analyses | Data description | Non-response bias? (Y= NRB, N= ROB) | Description of non-responders? | Results internally consistent? | Analysis results described in methods? | Conclusions justified by results? | Study limitations | Funding or COI? (Y= none; N= funding/COI) | Ethical approval | Total score (/20) |
| --- | --- | --- | --- | --- | --- | --- | --- | --- | --- | --- | --- | --- | --- | --- | --- | --- | --- | --- | --- | --- | --- |
| Decker 2022 | Y | Y | N | Y | Y | Y | N | Y | Y | Y | Y | Y | Y | N | Y | Y | Y | Y | N | Y | 16 |
| Holko 2022 | Y | Y | Y | Y | Y | Y | N | Y | Y | Y | Y | Y | Y | Y | Y | Y | Y | Y | N | Y | 18 |
| Varma 2022 | Y | Y | N | Y | Y | Y | N | Y | Y | Y | Y | Y | Y | N | Y | Y | Y | Y | N | Y | 16 |
| Paulides 2022 | Y | Y | N | Y | Y | Y | N | Y | Y | Y | Y | Y | N | N | Y | Y | Y | Y | Y | Y | 16 |
| Viazis 2022 | Y | Y | N | Y | Y | Y | N | Y | Y | Y | Y | Y | Y | - | Y | Y | Y | Y | N | Y | 16 |
| Paulides 2020 | Y | Y | Y | Y | Y | Y | N | Y | Y | Y | Y | Y | N | N | Y | Y | Y | Y | Y | Y | 17 |
| Yamabe 2019 | Y | Y | Y | Y | Y | Y | N | Y | Y | Y | Y | Y | Y | N | Y | Y | Y | Y | N | Y | 17 |
| Kawalec 2018 | Y | Y | N | Y | Y | Y | N | Y | Y | Y | Y | Y | Y | N | Y | Y | Y | Y | Y | - | 16 |
| vanGennep 2021 | Y | Y | N | Y | Y | Y | N | Y | Y | Y | Y | Y | N | N | Y | Y | Y | Y | Y | Y | 16 |
| Ruiz-Casas 2021 | Y | Y | N | Y | Y | Y | N | Y | Y | Y | Y | Y | N | N | Y | Y | Y | Y | N | Y | 15 |
| Rankala 2021 | Y | Y | N | Y | Y | Y | Y | Y | Y | Y | Y | Y | Y | Y | Y | Y | Y | Y | N | Y | 18 |
| Khalili 2020 | Y | Y | Y | Y | Y | Y | N | Y | Y | Y | Y | Y | Y | N | Y | Y | Y | Y | N | Y | 17 |
| Yu 2021 | Y | Y | N | Y | Y | Y | N | Y | Y | Y | Y | Y | Y | N | Y | Y | Y | Y | Y | Y | 17 |
| deSaBritoFroes 2021 | Y | Y | N | Y | Y | Y | N | Y | Y | Y | Y | Y | N | N | Y | Y | Y | Y | N | Y | 15 |
| Walter 2020 | Y | Y | N | Y | Y | Y | N | Y | Y | Y | Y | Y | - | N | Y | Y | Y | Y | N | Y | 15 |
| Manceur 2020 | Y | Y | N | Y | Y | Y | N | Y | Y | Y | Y | Y | - | N | Y | Y | Y | Y | Y | Y | 16 |
| Moon 2020 | Y | Y | N | Y | Y | Y | N | Y | Y | Y | Y | Y | Y | N | Y | Y | Y | Y | N | Y | 16 |
| Chao 2019 | Y | Y | N | Y | Y | Y | N | Y | Y | Y | Y | Y | Y | N | Y | Y | Y | Y | Y | Y | 17 |
| Parra 2019 | Y | Y | Y | Y | Y | Y | - | Y | Y | Y | Y | Y | - | - | Y | Y | Y | Y | N | Y | 16 |
| LeBerre 2019 | Y | Y | N | Y | Y | Y | N | Y | Y | Y | Y | Y | N | N | Y | Y | Y | Y | N | Y | 15 |
| Gonczi 2019 | Y | Y | N | Y | Y | Y | N | Y | Y | Y | Y | Y | - | - | Y | Y | Y | Y | N | Y | 15 |
| Everhov 2019 | Y | Y | N | Y | Y | Y | - | Y | Y | Y | Y | Y | - | - | Y | Y | Y | Y | N | Y | 15 |
| Everhov 2018 | Y | Y | N | Y | Y | Y | - | Y | Y | Y | Y | Y | - | - | Y | Y | Y | Y | N | Y | 15 |
| Williet 2017 | Y | Y | N | Y | Y | Y | N | Y | Y | Y | Y | Y | N | N | Y | Y | Y | Y | Y | - | 15 |
| Holko 2016 | Y | Y | Y | Y | Y | Y | N | Y | Y | Y | Y | Y | N | N | Y | Y | Y | Y | N | Y | 16 |
| DeBoer 2016 | Y | Y | N | Y | Y | Y | Y | Y | Y | Y | Y | Y | N | N | Y | Y | Y | Y | Y | Y | 17 |
| Zhou 2010 | Y | Y | N | Y | Y | Y | N | Y | Y | Y | Y | Y | Y | N | Y | Y | Y | Y | Y | Y | 17 |
| Boonen 2002 | Y | Y | N | Y | Y | Y | Y | Y | Y | Y | Y | Y | Y | Y | Y | Y | Y | Y | Y | - | 18 |
| Bernstein 2001 | Y | Y | N | Y | Y | Y | Y | Y | Y | Y | Y | Y | Y | Y | Y | Y | Y | Y | Y | Y | 19 |
| Wyke 1988 | Y | Y | N | Y | Y | Y | N | Y | Y | Y | Y | Y | Y | N | Y | Y | Y | N | Y | - | 15 |
| Sorensen 1987 | Y | Y | N | Y | Y | Y | Y | Y | Y | Y | Y | Y | Y | Y | Y | Y | Y | N | Y | - | 17 |
| Tiankanon 2021 | Y | Y | N | Y | Y | Y | - | Y | Y | Y | Y | Y | - | - | Y | Y | Y | Y | Y | Y | 16 |
| Ding 2022 | Y | Y | N | Y | Y | Y | N | Y | Y | Y | Y | Y | N | N | Y | Y | Y | Y | N | Y | 15 |
| Ganz 2016 | Y | Y | N | Y | Y | Y | - | Y | Y | Y | Y | Y | - | - | Y | Y | Y | Y | N | Y | 15 |
| Yan 2020 | Y | Y | N | Y | Y | Y | N | Y | Y | Y | Y | Y | N | N | Y | Y | Y | Y | N | Y | 15 |
| Ueno 2017 | Y | Y | N | Y | Y | Y | N | Y | Y | Y | Y | Y | N | N | Y | Y | Y | Y | N | Y | 15 |
| VanDerValk 2014 | Y | Y | Y | Y | Y | Y | Y | Y | Y | Y | Y | Y | Y | Y | Y | Y | Y | Y | N | Y | 19 |
| Hendrikson 1980 | Y | Y | N | Y | Y | Y | N | Y | Y | Y | Y | Y | N | N | Y | Y | Y | N | - | Y | 14 |
| Nurmi 2013 | Y | Y | N | Y | Y | Y | N | Y | Y | Y | Y | Y | N | N | Y | Y | Y | Y | Y | Y | 16 |
| Ghosh & Mitchell 2007 | Y | Y | N | Y | Y | Y | N | Y | Y | Y | Y | Y | N | N | Y | Y | Y | Y | N | - | 15 |
| Stjernman 2011 | Y | Y | N | Y | Y | Y | N | Y | Y | Y | Y | Y | Y | N | Y | Y | Y | Y | N | Y | 16 |
| Longobardi 2003 | Y | Y | N | Y | Y | Y | Y | Y | Y | Y | Y | Y | N | Y | Y | Y | Y | Y | N | - | 16 |
| Longobardi 2003 | Y | Y | N | Y | Y | Y | N | Y | Y | Y | Y | Y | Y | N | Y | Y | Y | Y | N | - | 15 |
| Mayberry 1992 | Y | Y | N | Y | Y | Y | N | Y | Y | Y | Y | Y | N | N | Y | Y | Y | Y | N | - | 14 |
| Gazzard 1978 | Y | Y | N | Y | Y | Y | - | Y | Y | Y | Y | Y | - | - | Y | Y | Y | N | - | - | 13 |
| Juan 2003 | Y | Y | N | Y | Y | Y | - | Y | Y | Y | Y | Y | - | - | Y | Y | Y | Y | N | Y | 15 |
| Blomqvist 1997 | Y | Y | N | Y | Y | Y | - | Y | Y | Y | Y | Y | - | - | Y | Y | Y | N | - | - | 13 |
| Mesterton 2009 | Y | Y | N | Y | Y | Y | N | Y | Y | Y | Y | Y | N | N | Y | Y | Y | Y | N | Y | 15 |
| Cross 2022 | Y | Y | N | Y | Y | Y | - | Y | Y | Y | Y | Y | - | - | Y | Y | Y | Y | N | Y | 15 |
| Viazis 2013 | Y | Y | N | Y | Y | Y | N | Y | Y | Y | Y | Y | Y | N | Y | Y | Y | Y | N | Y | 16 |
| Vaizey 2014 | Y | Y | N | Y | Y | Y | - | Y | Y | Y | Y | Y | - | - | Y | Y | Y | Y | N | Y | 15 |
| Ramos 2015 | Y | Y | N | Y | Y | y | N | Y | Y | Y | Y | Y | N | N | Y | Y | Y | Y | N | Y | 15 |
| Gibson 2014 | Y | Y | Y | Y | Y | Y | - | Y | Y | Y | Y | Y | - | - | Y | Y | Y | Y | N | Y | 16 |
| Lonnfors 2014 | Y | Y | N | Y | Y | Y | - | Y | Y | Y | Y | Y | - | - | Y | Y | Y | Y | N | - | 14 |

COI: Conflict of interest, NRB: no risk of bias, RB: risk of bias

| **Cohort studies** | **Research question** | **Study population definition** | **Participation rate >50%?** | **Participants selection** | **Sample size** | **Exposure measured prior to outcome?** | **Sufficient follow-up time?** | **Different levels of the exposure measured?** | **Exposures clearly defined?** | **Exposure assessed more than once?** | **Outcomes clearly defined?** | **Blinding** | **Loss to follow-up <20%?** | **Confounders adjusted for?** | **Quality Rating (Good, Fair, or Poor)** |
| --- | --- | --- | --- | --- | --- | --- | --- | --- | --- | --- | --- | --- | --- | --- | --- |
| Teich 2021 | Y | Y | Y | Y | Y | Y | Y | N | Y | Y | Y | N | Y | Y | Good |
| Topal 2020 | Y | Y | N/A | Y | N | N/A | Y | N | Y | N | Y | N/A | N/A | N | Fair |
| Willian 2018 | Y | Y | Y | Y | Y | Y | Y | N | Y | Y | Y | N | Y | Y | Good |
| Bessissow 2022 | Y | Y | Y | Y | Y | Y | Y | N | Y | Y | Y | N/A | N | N | Fair |
| Sciberras 2022 | Y | Y | NR | Y | Y | N | Y | Y | Y | N | Y | N/A | NR | Y | Fair |
| Toruner 2022 | Y | Y | Y | N/A | Y | Y | Y | Y | Y | Y | Y | N/A | Y | N | Good |
| West 2022 | Y | Y | Y | Y | N/A | Y | Y | Y | Y | Y | Y | N/A | Y | Y | Good |
| Gatopoulou 2021 | Y | Y | NR | Y | Y | Y | Y | Y | Y | Y | Y | NR | Y | N | Good |
| Teich 2020 | Y | Y | Y | Y | N | Y | Y | Y | Y | Y | Y | N/A | Y | N | Good |
| Christiansen 2019 | Y | Y | Y | Y | N | Y | Y | Y | Y | N | Y | N/A | Y | N | Good |
| Pillai 2019 | Y | Y | N/A | Y | Y | N | Y | Y | Y | N | Y | N/A | N/A | Y | Good |
| Loftus 2019 | Y | Y | N | Y | Y | Y | Y | Y | Y | Y | Y | NR | N | Y | Good |
| Travis 2017 | Y | Y | Y | Y | Y | Y | Y | Y | Y | Y | Y | N | Y | Y | Good |
| Spekhorst 2017 | Y | Y | Y | Y | Y | N | N | Y | Y | N | Y | N/A | N/A | Y | Fair |
| Kamat 2017 | Y | Y | Y | Y | Y | N | Y | Y | Y | N | Y | N | Y | Y | Fair |
| Aldeguer 2016 | Y | Y | Y | Y | Y | N | Y | Y | Y | Y | Y | N/A | Y | Y | Good |
| Vester-Andersen 2015 | Y | Y | Y | Y | Y | N | Y | Y | Y | N | Y | N/A | NR | Y | Fair |
| Zand 2015 | Y | Y | Y | Y | Y | Y | N/A | Y | Y | N | Y | N/A | Y | N | Fair |
| Cohen 2015 | Y | Y | NR | Y | Y | Y | Y | Y | Y | Y | Y | Y | Y | Y | Good |
| vanderHave 2015 | Y | Y | N | Y | Y | Y | Y | Y | Y | N | Y | N/A | Y | N | Fair |
| Yarlas 2015 | Y | Y | N | Y | Y | Y | Y | Y | Y | Y | Y | N | NR | Y | Good |
| Mandel 2014 | Y | Y | Y | Y | Y | N | Y | Y | Y | Y | Y | N/A | Y | N | Good |
| vanderValk 2014 | Y | Y | N | Y | N | Y | Y | N/A | Y | Y | Y | N | NR | Y | Fair |
| Siebert 2013 | Y | Y | NR | Y | N | Y | Y | N/A | Y | Y | Y | N | N | Y | Fair |
| Neovius 2013 | Y | Y | NR | Y | N | Y | Y | Y | Y | Y | Y | N | NR | N | Good |
| Gunnarsson 2013 | Y | Y | NR | Y | N | Y | Y | N/A | Y | Y | Y | N | NR | Y | Fair |
| Hoivik 2013 | Y | Y | Y | Y | N | Y | Y | N/A | Y | Y | Y | N | Y | Y | Good |
| Louis 2013 | Y | Y | Y | Y | N | Y | Y | Y | Y | Y | Y | N | Y | Y | Good |
| Benedini 2012 | Y | Y | Y | Y | N | Y | Y | N/A | Y | Y | Y | N | N | Y | Fair |
| Lichtiger 2010 | Y | Y | NR | Y | N | Y | Y | Y | Y | Y | Y | N | Y | Y | Good |
| Gibson 2008 | Y | Y | NR | Y | N | Y | Y | Y | Y | N | Y | N | N/A | N | Good |
| Stark 2006 | Y | Y | N | Y | N | Y | Y | N/A | Y | Y | Y | N | Y | Y | Fair |
| Bernklev 2006 | Y | Y | NR | Y | N | Y | Y | N/a | Y | Y | Y | N | NR | Y | Good |
| Armuzzi 2019 | Y | Y | NR | Y | N | Y | Y | N/a | Y | Y | Y | N | Y | Y | Good |
| Damgaard 1995 | Y | Y | NR | Y | N | Y | Y | N/A | Y | Y | Y | N | NR | N | Fair |
| Binder 1985 | Y | Y | Y | Y | N | Y | Y | N/A | Y | Y | Y | N | Y | Y | Good |
| Panaccione 2011 | Y | Y | NR | Y | N | Y | Y | Y | Y | Y | Y | N | Y | Y | Fair |
| **Case-control Studies** | Research question | Study population definition | Sample size | Controls & cases selection | Eligibility criteria | Cases and controls definitions | Cases and controls randomly selected from those eligible? | Use of concurrent controls? | Exposure occurred before outcome? | Valid measures of exposure? | Blinding | Confounders adjusted for? |  |  | **Quality Rating (Good, Fair, or Poor)** |
| Ananthakrishnan 2008 | Y | Y | N | Y | Y | Y | N/A | Y | Y | Y | N | Y |  |  | Good |

**Supplementary Table S4: Risk of bias of cohort and case-control studies as per the National Institutes of Health (NIH) tool**

N/A: Not applicable, NR: Not Reported

**Supplementary Table S5: Risk of bias of randomized clinical trials as per the Risk of Bias (ROB) tool**

| **Study** | **Randomization** | **Effect of assignment to intervention** | **Missing outcome data** | **Measurement of the outcome** | **Selection of the reported result** | **Overall Risk of Bias** |
| --- | --- | --- | --- | --- | --- | --- |
| Feagan 2010 | **+** | **+** | **?** | **+** | **+** | **?** |
| Reilly 2008 | **+** | **+** | **?** | **+** | **+** | **?** |
| Reinisch 2007 | **+** | **+** | **?** | **+** | **+** | **?** |
| Feagan 2005 | **+** | **+** | **+** | **+** | **+** | **+** |
